# Birnbaum Saunders distribution for imprecise data: statistical properties, estimation methods, and real life applications

**DOI:** 10.1038/s41598-024-57438-8

**Published:** 2024-03-23

**Authors:** Marwa K. Hassan, Muhammad Aslam

**Affiliations:** 1 Department of Mathematics, Faculty of Education, Ain Shams University, Cairo, 11566 Egypt; 2 Department of Statistics, Faculty of Science, King Abdulaziz University, 21551 Jeddah, Saudi Arabia

**Keywords:** Neutrosophic statistics, Simulation study, Birnbaum–Saunders distribution, Bayesian estimation, Maximum likelihood estimation, Engineering, Mathematics and computing

## Abstract

A neutrosophic statistic is a random variable and it has a neutrosophic probability distribution. So, in this paper, we introduce the new neutrosophic Birnbaum–Saunders distribution. Some statistical properties are derived, using Mathematica 13.1.1 and R-Studio Software. Two different estimation methods for parameters estimation are introduced for new distribution: maximum likelihood estimation method and Bayesian estimation method. A Monte-Carlo simulation study is used to investigate the behavior of parameters estimates of new distribution, compare the performance of different estimates, and compare between our distribution and the classical version of Birnbaum–Saunders. Finally, study the validity of our new distribution in real life.

## Introduction

Normal distribution is considered the most distribution used in our real life. Many new distributions are derived from normal distribution using different transformations. Two parameter Birnbaum–Saunders (BS) distribution is considered one of these distributions. ^1^introduced the BS distribution as a statistical model for fatigue life of structures under cyclic stress. In the recent years the BS distribution is used in many fields to its theoretical arguments associated with cumulative damage processes, its properties, and its relationship with the normal distribution. BS distribution is unimodal, positively skewed also it investigated for applications in engineering by many authors see^2–5^. Also, BS distribution has many applications in other fields such as business, environment and medicine see ^6–19^. Also, the BS distribution can be obtained as an approximation of inverse Gaussian (IG) distribution see^20^, it can see equal mixture of an inverse Gaussian and its reciprocal see^21^, Many statistical properties of BS distribution is studied by many authors such that probability density function pdf, hazard function (hf) because it plays an important role in lifetime data see^22–25^.

### Definition 1:

A random variable $X$ is said to be Birnbaum–Saunders distribution with shape parameter $\alpha >0$, scale parameter $\beta >0$ and denoted by $X\sim BS(\alpha ,\beta )$, if the probability density function (pdf) and the cumulative distribution (cdf) of $X$ are defined as follows respectively.

1 $$ f_{BS} \left( {x;\alpha ,\beta } \right) = \frac{1}{{\sqrt {2 \pi } }}\exp \left( {\frac{ - 1}{{2 \alpha^{2} }} \left( {\frac{x}{\beta } + \frac{\beta }{x} - 2} \right)} \right) \frac{{x^{{ - 3/2 \left( {x + \beta } \right)}} }}{2 \alpha \sqrt \beta },\quad x > 0, $$

2 $$ F_{BS} \left( {x;\alpha ,\beta } \right) = \frac{1}{2} \left( {1 + Erf\left( {\frac{\beta x - 1}{{\alpha \sqrt {2 \beta x} }}} \right)} \right). $$

where, $Erf$ is the error function.

**Figure 1:**
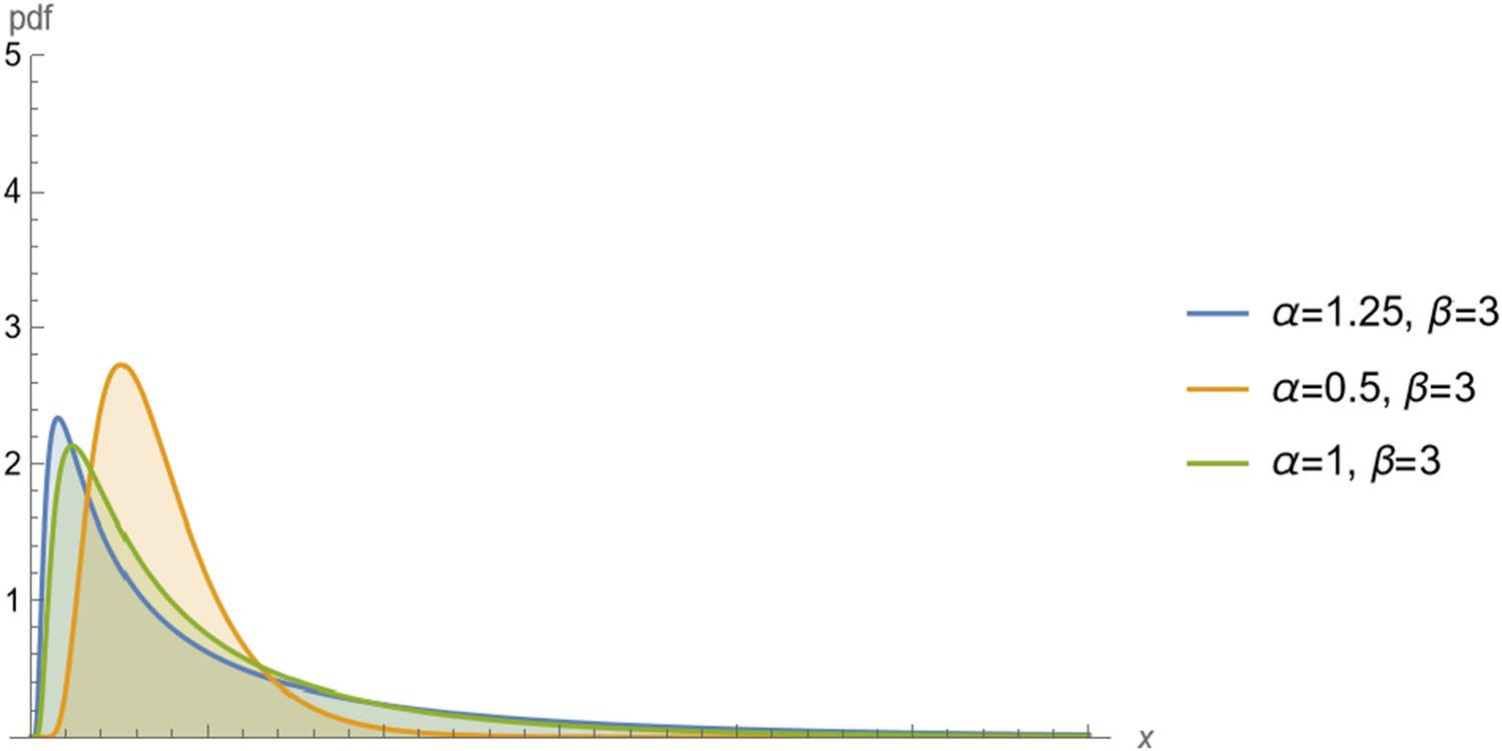
The pdf of $BS(\alpha ,\beta )$ for different values of shape parameter $\alpha$.

**Figure 2:**
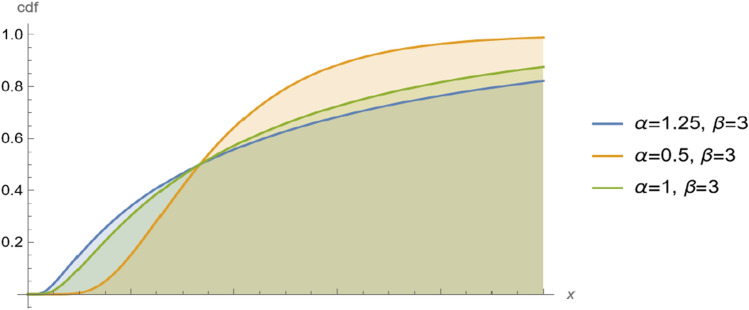
The cdf of $BS(\alpha ,\beta )$ for different values of shape parameter $\alpha$.

Figure 1 shows the pdf of $BS(\alpha ,\beta )$ for different values of shape parameter $\alpha$ we can the BS distribution is unimodal distribution. Also, Fig. 2 shows the cdf of $BS(\alpha ,\beta )$ for different values shape parameter $\alpha$. Two Figures show the changes in the distribution curve when the shape parameter takes different values.

The hazard function of BS distribution is defined as,

3 $$ \begin{aligned} hf_{BS} \left( {x;\alpha ,\beta } \right) & = \frac{{f_{BS} \left( {x;\alpha ,\beta } \right)}}{{1 - F_{BS} \left( {x;\alpha ,\beta } \right)}}, \\ & = \frac{{{\text{e}}^{{ - \frac{{\left( { - 1 + x\beta } \right)^{2} }}{{2x\alpha^{2} \beta }}}} \left( {1 + x\beta } \right)}}{{\sqrt {2\pi } \alpha \sqrt {x^{3} \beta } {\text{Erfc}}\left[ {\frac{ - 1 + x\beta }{{\sqrt 2 \alpha \sqrt {x\beta } }}} \right]}}. \\ \end{aligned} $$

**Figure 3:**
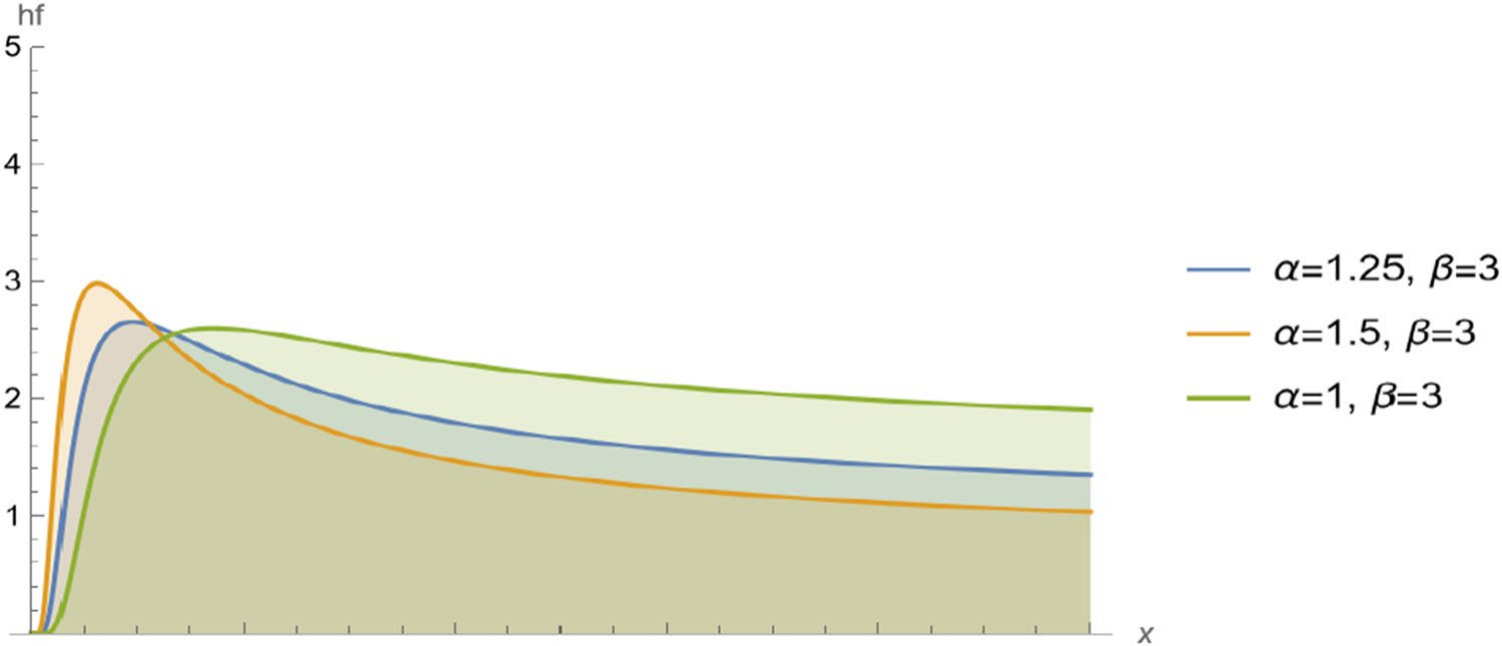
The hf of $BS(\alpha ,\beta )$ for different values of shape parameter $\alpha .$

Figure 3 shows the hf of $BS(\alpha ,\beta )$ for different values of shape parameter $\alpha .$ The Figure shows the changes in the distribution curve when the shape parameter takes different values.

The main reason of choosing BS distribution as a fatigue failure life distribution by ^1^ is known that for the analysis fatigue data used any two-parameter distribution such as Weibull, log-normal and gamma distributions. For importance of BS distribution, we proposed in this paper a new distribution called neutrosophic Birnbaum–Saunders distribution and denoted by $NBS({\alpha }_{N},{\beta }_{N}). NBS(\alpha ,\beta )$. In the literature of neutrosophic statistics, the start work in neutrosophic statistics is introduced by ^26^ when he showed that the neutrosophic logic is more efficient than fuzzy logic. Also, Smarandache^27^ present the neutrosophic statistics and showed also it is more efficient than classical statistics. Neuterosophic statistics is considered as the generalized of classical statistics and it is reduced to classical statistics when imprecise observations in the data. For the efficient of neutrosophic statistics see also^28–30^. Many authors introduced the neutrosophic probability distributions such as Poisson, exponential, binomial, normal, uniform, Weibull and so on see^27,31–35^ introduced the neutrosophic queueing theory in stochastic modeling.^36,37^ and^38^ investigated the neutrosophic time series. Recently, many authors studied the neutrosophic random variables see^39,40^ inserted the new notions on neutrosophic random variables. Granados and Sanabria^41^ studied independence neutrosophic random variables. Neutrosophic has many applications in many fields such as decision making, machine learning, intelligent disease diagnosis, communication services, pattern recognition, social network analysis and e-learning systems, physics, sequences spaces and so on for more details see^27,42–51^ and^52^. This paper is organized as follows, in section “Neutrosophic Birnbaum–Saunders distribution and its statistical properties”, we introduce the new distribution $NBS({\alpha }_{N},{\beta }_{N})$. and derived its statistical properties. In section “Parameter estimation”, Bayesian and non-Bayesian estimation methods are discussed to estimate the parameters of new distribution. In section “Simulation and comparative study”, the Monte-Carlo simulation and comparative study is performed to investigate the behavior of different estimates for the parameters of our distribution and compare between different estimates of parameters of new distribution. In section “Comparative study using real application”, real life data analysis is introduced. Finally, in section “Conclusion”, the conclusion of our study is introduced.

## Neutrosophic Birnbaum–Saunders distribution $NBS({\alpha }_{N},{\beta }_{N})$. and its statistical properties

In this section, we introduce the new distribution which called neutrosophic Birnbaum–Saunders distribution and denoted by $NBS({\alpha }_{N},{\beta }_{N})$. where $\alpha$ is a shape parameter and $\beta$ is the scale parameter. We use Mathematica 13.1 in all calculations in this section, for more details see^53^.

### Definition 2:

(The neutrosophic probability density function and neutrosophic cumulative distribution function of $NBS({\alpha }_{N},{\beta }_{N})$)

Let ${I}_{N}\in ({I}_{L},{I}_{U})$ be an indeterminacy interval, where $N$ is the neutrosophic statistical number and let ${X}_{N}={X}_{L}+{X}_{U} {I}_{N}$ be a random variable following neutrosophic Birnbaum–Saunders with scale parameter ${\beta }_{N}$ and shape parameter ${\alpha }_{N}$. If the neutrosophic probability density function $(npdf$) and neutrosophic cumulative distribution function $(ncdf)$ are defined as follows respectively,

4 $$ f\left( {x_{N} } \right) = \frac{{{\text{exp}}\left( {\frac{{ - \left( {\beta_{N} x_{Ni} - 1} \right)^{2} }}{{2 \alpha_{N}^{2} \beta_{N} x_{Ni} }}} \right)\beta_{N} \left( {1 + \beta_{N} x_{N} } \right)}}{{2\sqrt {2\pi } \alpha \sqrt {\beta_{N} x_{N}^{3} } }}\left( {1 + I_{N} } \right),\quad x_{N} > 0,\alpha_{N} > 0, \beta_{N} > 0. $$

5 $$ F\left( {X_{N} } \right) = \frac{1}{2}\left( {1 + {\text{Erf}}\left[ {\frac{{ - 1 + \beta_{N} x_{N} }}{{\sqrt 2 \alpha_{N} \sqrt {\beta_{N} x_{N} } }}} \right]} \right)\left( {1 + I_{N} } \right),\quad x_{N} > 0,\alpha_{N} > 0, \beta_{N} > 0. $$

**Figure 4:**
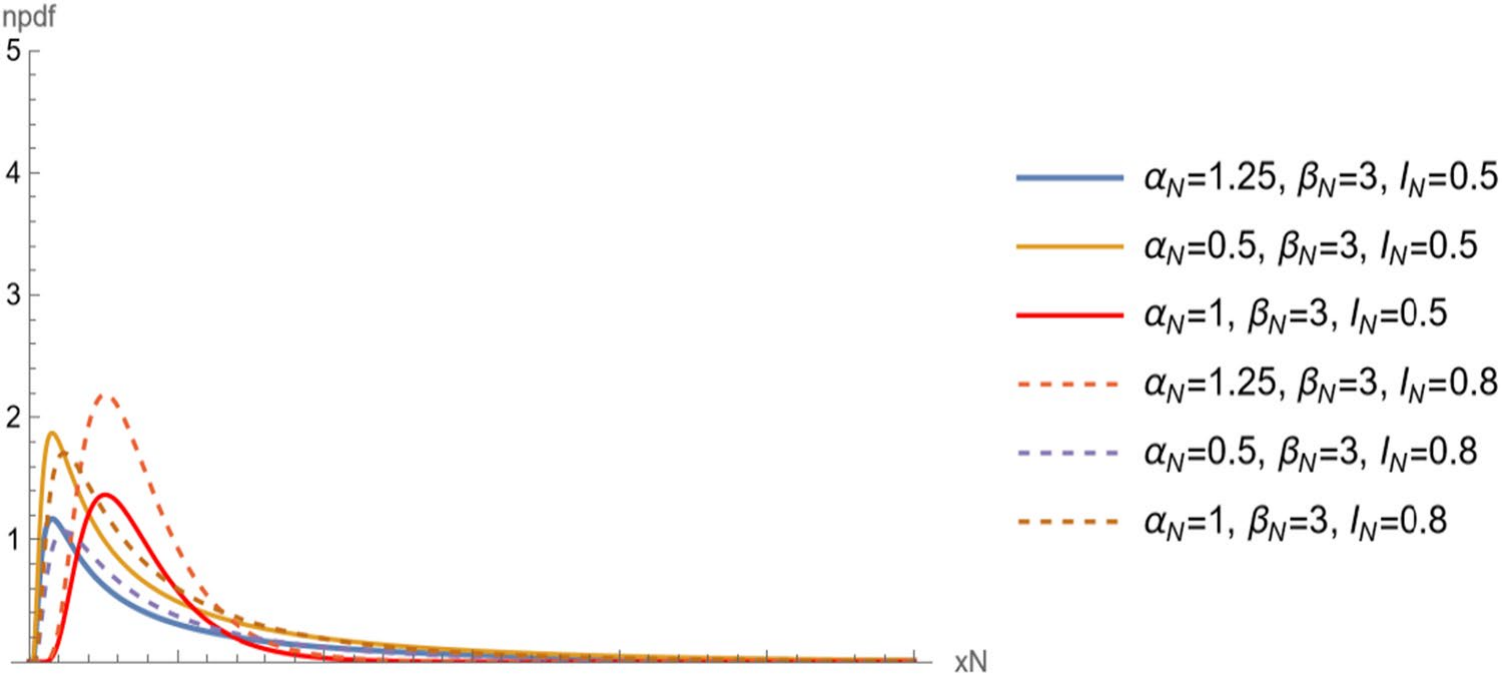
The $npdf$ for different values of ${\alpha }_{N}$ and ${\beta }_{N}$.

**Figure 5:**
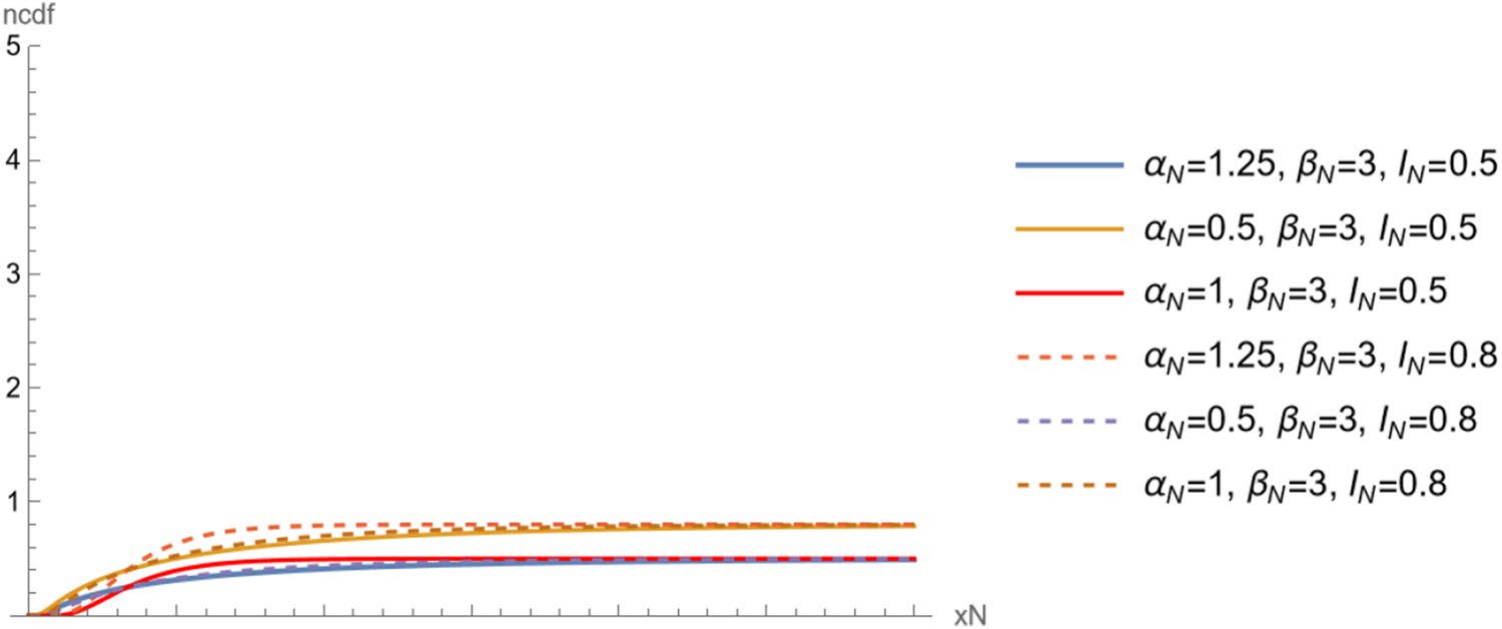
The $ncdf$ for different values of ${\alpha }_{N}$ and ${\beta }_{N}$.

Note that, the neutrosophic distribution go to the classical distribution when ${I}_{N}=0$. Figures 4 and 5 show $npdf$ and $ncdf$ for different values of ${\alpha }_{N}$ and ${\beta }_{N}.$ Two show the changes in the distribution curve when the shape parameter takes different values. Also we can see the effect of indeterminacy parameter on curves.

### Definition 3:

(The neutrosophic reliability function and neutrosophic hazard function of $NBS({\alpha }_{N},{\beta }_{N})$)

**Figure 6:**
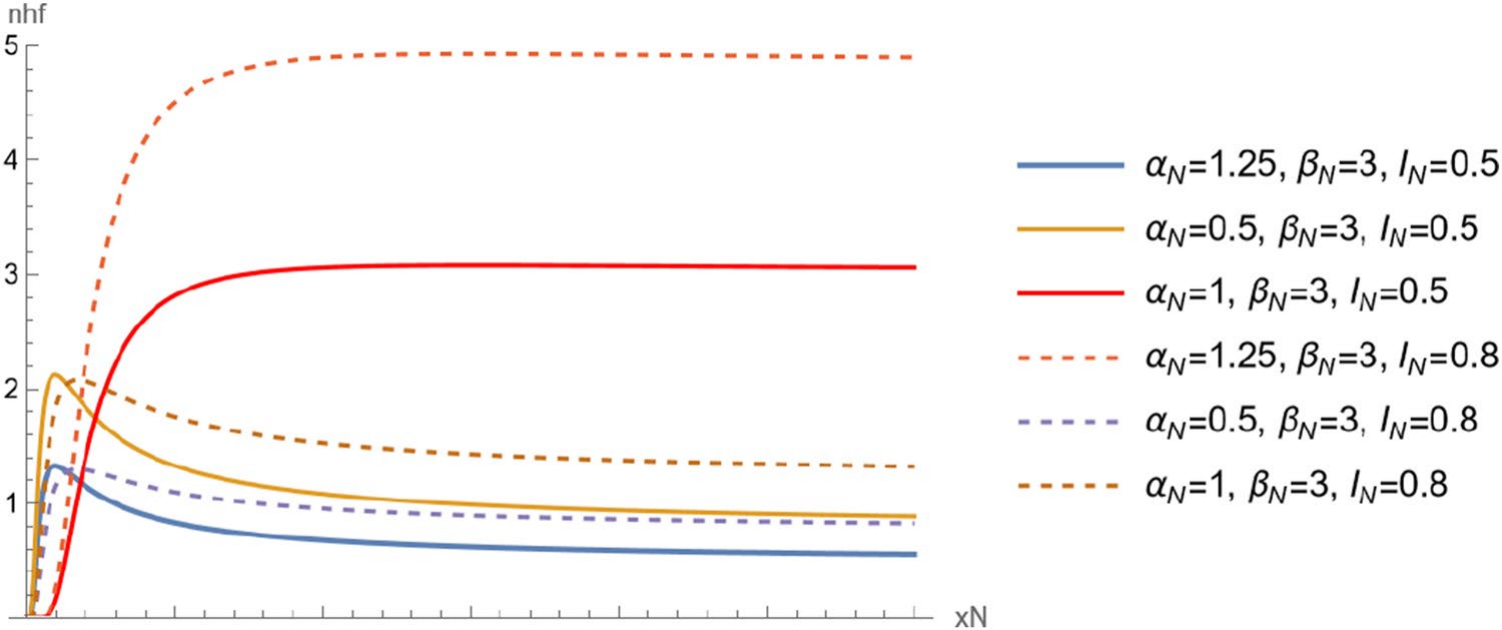
The nhf for different values of ${\alpha }_{N}$ and ${\beta }_{N}$.

The neutrosophic reliability function of ${X}_{N}$ is a random variable following neutrosophic Birnbaum–Saunders with scale parameter ${\beta }_{N}$ and shape parameter ${\alpha }_{N}$ is defined as,

6 $$ \begin{aligned} R\left( {x_{N} } \right) & = 1 - F\left( {x_{N} } \right), \\ & = 1 - \frac{ 1}{2}\left( {1 + {\text{Erf}}\left[ {\frac{{ - 1 + \beta_{N} x_{N} }}{{\sqrt 2 \alpha_{N} \sqrt {\beta_{N} x_{N} } }}} \right]} \right)\left( {1 + I_{N} } \right),\quad x_{N} > 0, \alpha_{N} > 0,\beta_{N} > 0. \\ \end{aligned} $$

and the neutrosophic hazard function of ${X}_{N}$ is defined as,

7 $$ h\left( {x_{N} } \right) = \left( {\left( {{\text{e}}^{{ - \frac{{\left( { - \frac{1}{{\sqrt {\beta_{N} x_{N} } }} + \sqrt {\beta_{N} x_{N} } } \right)^{2} }}{{2\alpha_{N}^{2} }}}} \left( {1 + \beta_{N} x_{N} } \right)} \right)/\left( {\sqrt {2\pi } \alpha {\text{Erfc}}\left[ {\frac{{ - \frac{1}{{\sqrt {\beta_{N} x_{N} } }} + \sqrt {\beta_{N} x_{N} } }}{{\sqrt 2 \alpha_{N} }}} \right]\sqrt {\beta_{N} x_{N}^{3} } } \right)} \right)\left( {1 + I_{N} } \right). $$

and denoted by $nhf$. Figure 6 shows the $nhf$ for different values of ${\alpha }_{N}$ and ${\beta }_{N}.$ The Figure show the changes in the distribution curve when the shape parameter takes different values. Also we can see the effect of indeterminacy parameter on curves.

Now,we discuss some statistical properties of new proposed distribution $NBS({\alpha }_{N},{\beta }_{N})$ such as mode, median, moments, moment generating function, quantile function, order statistics, entropy.

I. Mode:

To find the mode of neutrosophic Birnbaum- Saunders distribution solve the following nonlinear equation with respect to ${x}_{N}$,

$$ \frac{{{\text{exp}}\left( {\frac{{ - \left( {\beta_{N} x_{Ni} - 1} \right)^{2} }}{{2 \alpha_{N}^{2} \beta_{N} x_{Ni} }}} \right)\left( {1 + I_{N} } \right)x_{N} \left( { - 1 + \beta_{N} x_{N} \left( { - 1 + 3\alpha_{N}^{2} + \beta_{N} x_{N} \left( {1 + \alpha_{N}^{2} + \beta_{N} x_{N} } \right)} \right)} \right)}}{{4\sqrt {2\pi } \alpha_{N}^{3} \left( {\beta_{N} x_{N}^{3} } \right)^{3/2} }} = 0. $$

Then, the mode at ${x}_{N}={\text{Root}}[-1+(-{\beta }_{N}+3{{\alpha }_{N}}^{2}\beta )\#1+({{\beta }_{N}}^{2}+{{\alpha }_{N}}^{2}{{\beta }_{N}}^{2}){\#1}^{2}+{{\beta }_{N}}^{3}{\#1}^{3}\&,1]$

Where, $$0<{\alpha }_{N}<{\text{Root}}[-64+64{\#1}^{2}-92{\#1}^{4}+9{\#1}^{6}\&,\mathrm{2,0}]\&\&{\beta }_{N}>0)||({\text{Root}}[-64+64{\#1}^{2}-92{\#1}^{4}+9{\#1}^{6}\&,\mathrm{1,0}]<{\alpha }_{N}<0\&\&{\beta }_{N}>0)||{\beta }_{N}<0$$.

When $${\alpha }_{N}=1.5, \beta =2$$ then $${x}_{N}=0.0794$$.

**II. Median**

The median of $NBS({\alpha }_{N},{\beta }_{N})$ is given by.

$$P\left[{X}_{N}<m\right]={\int }_{0}^{m}f\left({x}_{N}\right)d{x}_{N}=0.5$$

$$\frac{1}{2}(1+{\text{Erf}}[\frac{-1+{\beta }_{N}\mathrm{ m}}{\sqrt{2}{\alpha }_{N}\sqrt{{\beta }_{N}\mathrm{ m}}}])(1+{I}_{N})=0.5$$

$$ \begin{aligned} m & = \left( {\frac{{0.5\left( { - \beta_{N} \left( { - 2 - 2\alpha_{N}^{2} {\text{InverseErf}}\left[ { - \frac{{I_{N} }}{{1 + I_{N} }}} \right]^{2} } \right) - \sqrt { - 4\beta_{N}^{2} + \beta_{N}^{2} \left( { - 2 - 2\alpha_{N}^{2} {\text{InverseErf}}\left[ { - \frac{{I_{N} }}{{1 + I_{N} }}} \right]^{2} } \right)^{2} } } \right)}}{{\beta_{N}^{2} }},} \right. \\ & \quad \left. {\frac{{0.5\left( { - \beta_{N} \left( { - 2 - 2\alpha_{N}^{2} {\text{InverseErf}}\left[ { - \frac{{I_{N} }}{{1 + I_{N} }}} \right]^{2} } \right) + \sqrt { - 4\beta_{N}^{2} + \beta_{N}^{2} \left( { - 2 - 2\alpha_{N}^{2} {\text{InverseErf}}\left[ { - \frac{{I_{N} }}{{1 + I_{N} }}} \right]^{2} } \right)^{2} } } \right)}}{{\beta_{N}^{2} }}} \right). \\ \end{aligned} $$

When $${\alpha }_{N}=1.5, {\beta }_{N}=2, { I}_{N}=0.2$$ then $$m=(\mathrm{0.3651,0.6846})$$.

**III. r-th moments of origin**

The r-th moments of origin of $NBS({\alpha }_{N},{\beta }_{N})$ is defined as

$$ \begin{aligned} E\left[ {X_{N}^{r} } \right] & = \mathop \smallint \limits_{0}^{\infty } x_{N}^{r} f\left( {x_{N} } \right)dx_{N} , \\ & = \frac{{{\text{exp}}\left( {1/\alpha_{N}^{2} } \right)\sqrt {\beta_{N} } \left( {\frac{{\beta_{N} }}{{\alpha_{N}^{2} }}} \right)^{{ - \frac{5}{4} - \frac{r}{2}}} \left( {\alpha_{N}^{2} \beta_{N} } \right)^{{ - \frac{1}{4} - \frac{r}{2}}} \left( {\beta_{N} {\text{BesselK}}\left[ { - \frac{1}{2} - r,\frac{{\sqrt {\frac{{\beta_{N} }}{{\alpha_{N}^{2} }}} }}{{\sqrt {\alpha_{N}^{2} \beta_{N} } }}} \right] + \sqrt {\frac{{\beta_{N} }}{{\alpha_{N}^{2} }}} \sqrt {\alpha_{N}^{2} \beta_{N} } {\text{BesselK}}\left[ {\frac{1}{2} - r,\frac{{\sqrt {\frac{{\beta_{N} }}{{\alpha_{N}^{2} }}} }}{{\sqrt {\alpha_{N}^{2} \beta_{N} } }}} \right]} \right)\left( {1 + I_{N} } \right)}}{{\sqrt {2\pi } \alpha_{N}^{3} }}. \\ \end{aligned} $$

**IV. Mean**

The mean of $NBS({\alpha }_{N},{\beta }_{N})$ is given by,

$$ \begin{aligned} E\left[ {X_{N} } \right] & = \mathop \smallint \limits_{0}^{\infty } x_{N} f\left( {x_{N} } \right)dx_{N} , \\ & = \frac{{ {\text{exp}}\left( {\beta_{N} - \sqrt {\frac{{\beta_{N} }}{{\alpha_{N}^{2} }}} \sqrt {\alpha_{N}^{2} \beta_{N} } /\alpha_{N}^{2} \beta_{N} } \right) \left( {\frac{{\left( {1 + \alpha_{N}^{2} } \right)\beta_{N} }}{{\sqrt {\frac{{\beta_{N} }}{{\alpha_{N}^{2} }}} }} + \sqrt {\alpha_{N}^{2} \beta_{N} } } \right)\left( {1 + I_{N} } \right)}}{{2\alpha_{N} \beta_{N}^{3/2} }}. \\ \end{aligned} $$

**V. Variance**

The Variance of $NBS({\alpha }_{N},{\beta }_{N})$ is given by.

$$ \begin{aligned} Var\left[ {X_{N} } \right] & = E\left[ {X_{N}^{2} } \right] - \left( {E\left[ {X_{N} } \right]} \right)^{2} , \\ & = \frac{{\exp \left( {\beta_{N} - \frac{{\sqrt {\frac{{\beta_{N} }}{{\alpha_{N}^{2} }}} \sqrt {\alpha_{N}^{2} \beta_{N} } }}{{\alpha_{N}^{2} \beta_{N} }}} \right)\alpha_{N} \left( {\beta_{N} + 3\alpha_{N}^{2} \beta_{N} + \left( {1 + \alpha_{N}^{2} + 3\alpha_{N}^{4} } \right)\sqrt {\frac{{\beta_{N} }}{{\alpha^{2} }}} \sqrt {\alpha^{2} \beta_{N} } } \right)\left( {1 + I_{N} } \right)}}{{2\beta^{5/2} \sqrt {\alpha^{2} \beta } }} \\ & \quad - \frac{{\exp (\left( {2\beta_{N} - 2\sqrt {\alpha_{N}^{2} \beta_{N} } \sqrt { - 2t + \frac{{\beta_{N} }}{{\alpha_{N}^{2} }}} } \right))/\alpha_{N}^{2} \beta_{N} )\left( {\beta_{N} + \sqrt {\alpha_{N}^{2} \beta_{N} } \sqrt { - 2t + \frac{{\beta_{N} }}{{\alpha_{N}^{2} }}} } \right)^{2} \left( {1 + I_{N} } \right)^{2} }}{{4\beta_{N} \left( { - 2t\alpha_{N}^{2} + \beta_{N} \beta } \right)}} \\ \end{aligned} $$

**VI. Moment generating function**

The moment generating function of $NBS({\alpha }_{N},{\beta }_{N})$ is given by,

$$ \begin{aligned} M_{{X_{N} }} \left( t \right) & = E\left[ {e^{{t X_{N} }} } \right], \\ & = \frac{{{\text{exp}}\left( {\left( {\beta_{N} - \sqrt {\alpha_{N}^{2} \beta_{N} } \sqrt { - 2t + \frac{{\beta_{N} }}{{\alpha_{N}^{2} }}} } \right)/\alpha_{N}^{2} \beta_{N} } \right)\left( {\beta_{N} + \sqrt {\alpha_{N}^{2} \beta_{N} } \sqrt { - 2t + \frac{{\beta_{N} }}{{\alpha_{N}^{2} }}} } \right)\left( {1 + I_{N} } \right)}}{{2\alpha_{N} \sqrt {\beta_{N} } \sqrt { - 2t + \frac{{\beta_{N} }}{{\alpha_{N}^{2} }}} }}. \\ \end{aligned} $$

**VII. Characteristic function**

The characteristic function of $NBS({\alpha }_{N},{\beta }_{N})$ is given by,

$$ \begin{aligned} {\Phi }_{{X_{N} }} \left( t \right) & = E\left[ {e^{{i t X_{N} }} } \right], \\ & = \frac{{ {\text{exp}}\left( {\left( {\beta_{N} - \sqrt {\alpha_{N}^{2} \beta_{N} } \sqrt { - 2it + \frac{{\beta_{N} }}{{\alpha_{N}^{2} }}} } \right)/\alpha_{N}^{2} \beta_{N} } \right)\left( {1 + I_{N} } \right)\left( {\beta_{N} + \sqrt {\alpha_{N}^{2} \beta_{N} } \sqrt { - 2it + \frac{{\beta_{N} }}{{\alpha_{N}^{2} }}} } \right)}}{{2\alpha_{N} \sqrt {\beta_{N} } \sqrt { - 2it + \frac{{\beta_{N} }}{{\alpha_{N}^{2} }}} }}. \\ \end{aligned} $$

where $$i=\sqrt{-1}$$.

**VIII. Cumulant generating function**

The cumulant generating function of $NBS({\alpha }_{N},{\beta }_{N})$ is given by.

$$ \begin{aligned} C_{{X_{N} }} \left( t \right) & = {\text{Log}}[{\Phi }_{{X_{N} }} \left( t \right)], \\ & = Log\left[ {\frac{{{\text{exp}}\left( {\left( {\beta_{N} - \sqrt {\alpha_{N}^{2} \beta_{N} } \sqrt { - 2it + \frac{{\beta_{N} }}{{\alpha_{N}^{2} }}} } \right)\backslash \alpha_{N}^{2} \beta_{N} } \right)\left( {1 + I_{N} } \right)\left( {\beta_{N} + \sqrt {\alpha_{N}^{2} \beta_{N} } \sqrt { - 2it + \frac{{\beta_{N} }}{{\alpha_{N}^{2} }}} } \right)}}{{2\alpha_{N} \sqrt {\beta_{N} } \sqrt { - 2it + \frac{{\beta_{N} }}{{\alpha_{N}^{2} }}} }}} \right]. \\ \end{aligned} $$

where $$i=\sqrt{-1}.$$

**IX. Quantile function**

The quantile function of $NBS({\alpha }_{N},{\beta }_{N})$ is given by.

$$F\left({Q}_{{X}_{N}}\left(p\right)\right)=P,$$

$$ P = \left( {\frac{{2 \beta_{N} \left( {1 + k^{2} \alpha_{N}^{2} } \right) - \sqrt {4 \beta_{N}^{2} \left( {1 + k^{2} \alpha_{N}^{2} } \right) - 4\beta_{N}^{2} } }}{{2\beta_{N}^{2} }}, \frac{{2 \beta_{N} \left( {1 + k^{2} \alpha_{N}^{2} } \right) + \sqrt {4 \beta_{N}^{2} \left( {1 + k^{2} \alpha_{N}^{2} } \right) - 4\beta_{N}^{2} } }}{{2\beta_{N}^{2} }}} \right) $$

where, $$k^{2} = {\text{InverseErf}}\left[ {\frac{{2 P - 1 - I_{N} }}{{1 + I_{N} }}} \right]^{2}$$.

**X. Order statistics**

For given any random variables ${X}_{N1}\dots {X}_{NN}$, the order statistics. ${X}_{N(1)}\dots {X}_{N(N)}$ are also random variables, defined by sorting the values of ${X}_{N1}\dots {X}_{NN}$ in increasing order. For a random sample ${X}_{N(1)}\dots {X}_{N(N)}$ the npdf ${f}_{{X}_{N}\left(r\right)}\left({x}_{N}\right)$ and ncdf ${F}_{{X}_{N}\left(r\right)}\left({x}_{N}\right)$ are defined as follows:

$$ \begin{aligned} f_{{X_{N} \left( r \right)}} \left( {x_{N} } \right) & = \frac{n!}{{\left( {r - 1} \right)!\left( {n - r} \right)!}} f_{{X_{N} }} \left( {x_{N} } \right) \left[ {F_{{X_{N} }} \left( {x_{N} } \right)} \right]^{r - 1} \left[ {1 - F_{{X_{N} }} \left( {x_{N} } \right)} \right]^{n - r} , \\ & = \frac{n!}{{\left( {r - 1} \right)!\left( {n - r} \right)!}} \left( {\frac{{{\text{exp}}\left( {\frac{{ - \left( {\beta_{N} x_{Ni} - 1} \right)^{2} }}{{2 \alpha_{N}^{2} \beta_{N} x_{Ni} }}} \right)\beta_{N} \left( {1 + \beta_{N} x_{N} } \right)}}{{2\sqrt {2\pi } \alpha \sqrt {\beta_{N} x_{N}^{3} } }}\left( {1 + I_{N} } \right)} \right) \\ & \quad \left( {\frac{1}{2}\left( {1 + {\text{Erf}}\left[ {\frac{{ - 1 + \beta_{N} x_{N} }}{{\sqrt 2 \alpha_{N} \sqrt {\beta_{N} x_{N} } }}} \right]} \right)\left( {1 + I_{N} } \right)} \right)^{r - 1} \left( {1 - \frac{1}{2}\left( {{\text{Erf}}\left[ {\frac{{ - 1 + \beta_{N} x_{N} }}{{\sqrt 2 \alpha_{N} \sqrt {\beta_{N} x_{N} } }}} \right]} \right)\left( {1 + I_{N} } \right)} \right)^{n - r} . \\ \end{aligned} $$

$$ \begin{aligned} F_{{X_{N} \left( r \right)}} \left( {x_{N} } \right) & = \mathop \sum \limits_{j = r}^{n} \left( {\begin{array}{*{20}c} n \\ j \\ \end{array} } \right) \left[ {F_{{X_{N} }} \left( {x_{N} } \right)} \right]^{j} \left[ {1 - F_{{X_{N} }} \left( {x_{N} } \right)} \right]^{n - j} , \\ & = \mathop \sum \limits_{j = r}^{n} \left( {\begin{array}{*{20}c} n \\ j \\ \end{array} } \right)\left( {\frac{1}{2}\left( {1 + {\text{Erf}}\left[ {\frac{{ - 1 + \beta_{N} x_{N} }}{{\sqrt 2 \alpha_{N} \sqrt {\beta_{N} x_{N} } }}} \right]} \right)\left( {1 + I_{N} } \right)} \right)^{j} \\ & \quad \left( {1 - \frac{1}{2}\left( {1 + {\text{Erf}}\left[ {\frac{{ - 1 + \beta_{N} x_{N} }}{{\sqrt 2 \alpha_{N} \sqrt {\beta_{N} x_{N} } }}} \right]} \right)\left( {1 + I_{N} } \right)} \right)^{n - j} . \\ \end{aligned} $$

**XI. Entropy**

Entropy is considered one of the most popular measures of uncertainty.^54^ introduced the differential entropy $H(X)$ as follows:

$$H\left(X\right)=-{\int }_{-\infty }^{\infty }f\left(x\right) Log\left(f\left(x\right)\right)dx.$$

Rényi^55^ introduced Renyi entropy which finds its source in the information theory. He defined the Renyi entropy as follow:

$${I}_{\delta }\left(X\right)=\frac{1}{1-\delta } Log\left[{\int }_{-\infty }^{\infty }{\left(f\left(x\right)\right)}^{\delta } dx\right].$$

Where, $$\delta \ne 1$$ and $$\delta >0.$$

Tsallis^56^ introduced q-entropy which comes from statistical physics. He defined the q-entropy as follows:

$$ H_{\delta } \left( X \right) = \frac{1}{1 - q}{ }\left( {1 - \mathop \smallint \limits_{ - \infty }^{\infty } \left( {f\left( x \right)} \right)^{q} { }dx} \right). $$

Where, $q\ne 1$ and $q>0.$ Now, the three entropies are defined for $NBS({\alpha }_{N},{\beta }_{N})$ as follows:

$$ \begin{aligned} H\left( {X_{N} } \right) & = - Log\left[ k \right] - \frac{3}{2} \left( {\left( {\left( {{\text{exp}}\left( {\left( {\beta_{N} - \sqrt {\frac{{\beta_{N} }}{{\alpha_{N}^{2} }}} \sqrt {\alpha_{N}^{2} \beta_{N} } } \right)\backslash \alpha_{N}^{2} \beta_{N} } \right)\left( {1 + I_{N} } \right)\sqrt {\beta_{N} } } \right.} \right.} \right. \\ & \quad \left( {\sqrt {2\pi } \alpha_{N}^{2} \sqrt {\frac{{\sqrt {\alpha_{N}^{2} \beta_{N} } }}{{\alpha_{N}^{4} \sqrt {\frac{{\beta_{N} }}{{\alpha_{N}^{2} }}} }}} \left( {\beta_{N} + \sqrt {\frac{{\beta_{N} }}{{\alpha_{N}^{2} }}} \sqrt {\alpha_{N}^{2} \beta_{N} } } \right) \left( {{\text{Log}}\left[ {\frac{{\beta_{N} }}{{\alpha_{N}^{2} }}} \right] + {\text{Log}}\left[ {\alpha_{N}^{2} \beta_{N} } \right]} \right)} \right. \\ & \quad + \left( {4 {\text{exp}}\left( {\sqrt {\alpha_{N}^{2} \beta_{N} } /\alpha_{N}^{4} \sqrt {\frac{{\beta_{N} }}{{\alpha_{N}^{2} }}} } \right)} \right)\beta {\text{BesselK}}^{{\left( {1,0} \right)}} \left[ { - \frac{1}{2},\frac{{\sqrt {\frac{{\beta_{N} }}{{\alpha_{N}^{2} }}} }}{{\sqrt {\alpha_{N}^{2} \beta_{N} } }}} \right] + {\text{exp}}\left( {\sqrt {\alpha_{N}^{2} \beta_{N} } /\alpha_{N}^{4} \sqrt {\frac{{\beta_{N} }}{{\alpha_{N}^{2} }}} } \right)\sqrt {\frac{{\beta_{N} }}{{\alpha_{N}^{2} }}} \\ & \quad \left. {\left. {\left. {\left. {\sqrt {\alpha_{N}^{2} \beta_{N} } {\text{BesselK}}^{{\left( {1,0} \right)}} \left[ {\frac{1}{2},\frac{{\sqrt {\frac{{\beta_{N} }}{{\alpha_{N}^{2} }}} }}{{\sqrt {\alpha_{N}^{2} \beta_{N} } }}} \right]} \right)} \right)/\left( {4\sqrt {2\pi } \alpha_{N}^{3} \left( {\frac{{\beta_{N} }}{{\alpha_{N}^{2} }}} \right)^{5/4} \left( {\alpha_{N}^{2} \beta_{N} } \right)^{1/4} } \right)} \right)} \right) \\ & \quad - \frac{{\left( {1 + I_{N} } \right) ( {\text{exp}}\left( {\left( {\beta_{N} - \sqrt {\frac{{\beta_{N} }}{{\alpha_{N}^{2} }}} \sqrt {\alpha_{N}^{2} \beta_{N} } } \right)\backslash \alpha_{N}^{2} \beta_{N} } \right)\left( {\frac{{\beta_{N} }}{{\sqrt {\frac{{\beta_{N} }}{{\alpha_{N}^{2} }}} }} + \sqrt {\alpha_{N}^{2} \beta_{N} } } \right)}}{{4\alpha_{N} \sqrt {\beta_{N} } }} - \mathop \smallint \limits_{ - \infty }^{\infty } Log\left[ {1 - \beta x_{N} } \right]f\left( x \right)dx, \\ \end{aligned} $$

$$\begin{aligned} I_{\delta } \left( {X_{N} } \right) & = \frac{1}{1 - \delta }Log\left[ {k^{\delta } \mathop \sum \limits_{c = 0}^{\delta } \left( {\begin{array}{*{20}c} \delta \\ c \\ \end{array} } \right) \frac{{\beta_{N}^{c} }}{{\sqrt {2\pi } \alpha_{N} \sqrt {\beta_{N} } }}\left( {{\text{exp}}\left( {2/\alpha_{N}^{2} } \right)\left( {\frac{{\beta_{N} }}{{\alpha_{N}^{2} }}} \right)^{{\frac{1}{4}\left( { - 1 - 2c + 3\delta } \right)}} \left( {\alpha_{N}^{2} \beta_{N} } \right)^{{\frac{1}{4}\left( { - 1 - 2c + 3\delta } \right)}} } \right.} \right. \\ & \quad \left. {\left. {\left( {\beta {\text{BesselK}}\left[ {\frac{1}{2}\left( { - 1 - 2c + 3\delta } \right),\frac{{2\sqrt {\frac{{\beta_{N} }}{{\alpha_{N}^{2} }}} }}{{\sqrt {\alpha_{N}^{2} \beta_{N} } }}} \right] + \sqrt {\frac{{\beta_{N} }}{{\alpha_{N}^{2} }}} \sqrt {\alpha_{N}^{2} \beta_{N} } {\text{BesselK}}\left[ {\frac{1}{2}\left( {1 - 2c + 3\delta } \right),\frac{{2\sqrt {\frac{{\beta_{N} }}{{\alpha_{N}^{2} }}} }}{{\sqrt {\alpha_{N}^{2} \beta_{N} } }}} \right]} \right)\left( {1 + I_{N} } \right)} \right)} \right] \\ \end{aligned}$$where, $$k=\frac{1+{I}_{N}}{2 {\alpha }_{N} \sqrt{2 {\beta }_{N} \pi }}.$$

$$ \begin{aligned} H_{Q} \left( {X_{N} } \right) & = \frac{1}{1 - q} \left( {1 - \left( {k^{q} \mathop \sum \limits_{c = 0}^{q} \left( {\begin{array}{*{20}c} q \\ c \\ \end{array} } \right) \frac{{\beta_{N}^{c} }}{{\sqrt {2\pi } \alpha_{N} \sqrt {\beta_{N} } }}} \right.} \right. \\ & \quad \left( {{\text{exp}}\left( {2/\alpha_{N}^{2} } \right)\left( {\frac{{\beta_{N} }}{{\alpha_{N}^{2} }}} \right)^{{\frac{1}{4}\left( { - 1 - 2c + 3q} \right)}} \left( {\beta {\text{BesselK}}\left[ {\frac{1}{2}\left( { - 1 - 2c + 3q} \right),\frac{{2\sqrt {\frac{{\beta_{N} }}{{\alpha_{N}^{2} }}} }}{{\sqrt {\alpha_{N}^{2} \beta_{N} } }}} \right]} \right.} \right. \\ & \quad \left. {\left. {\left. { + \sqrt {\frac{{\beta_{N} }}{{\alpha_{N}^{2} }}} \sqrt {\alpha_{N}^{2} \beta_{N} } {\text{BesselK}}\left[ {\frac{1}{2}\left( {1 - 2c + 3q} \right),\frac{{2\sqrt {\frac{{\beta_{N} }}{{\alpha_{N}^{2} }}} }}{{\sqrt {\alpha_{N}^{2} \beta_{N} } }}} \right]} \right)\left( {1 + I_{N} } \right)} \right)} \right). \\ \end{aligned} $$

where, $$k=\frac{1+{I}_{N}}{2 {\alpha }_{N} \sqrt{2 {\beta }_{N} \pi }}.$$

## Parameter estimation

In this section, maximum likelihood and Bayesian estimation methods were used to estimate the parameters of our new proposed distribution neutrosophic Birnbaum–Saunders distribution.

### Maximum likelihood estimation method

Let ${X}_{N1}\dots {X}_{Nn}$ be a random sample from $NBS({\alpha }_{N},{\beta }_{N})$. Then the likelihood function is given by.

$$\begin{aligned} L\left( {x_{Ni} ;\alpha_{N} ,\beta_{N} } \right) & = \mathop \prod \limits_{i = 1}^{n} f\left( {x_{Ni} ;\alpha_{N} ,\beta_{N} } \right). \\ & = \mathop \prod \limits_{i = 1}^{n} \frac{{\left( {1 + I_{N} } \right)}}{{2 \alpha_{N} \sqrt {2 \pi x_{Ni}^{3} } }} \left( {1 + \beta_{N} x_{Ni} } \right)\exp \left( {\frac{{ - \left( {\beta_{N} x_{Ni} - 1} \right)^{2} }}{{2 \alpha_{N}^{2} \beta_{N} x_{Ni} }}} \right), \\ \end{aligned}$$

and its corresponding log-likelihood function is given by,

$$ \begin{aligned} logL\left( {x_{Ni} ;\alpha_{N} ,\beta_{N} } \right) & = n\left( {\log \left( {1 + I_{N} } \right) - \log \left( 2 \right) - \frac{1}{2}\log \left( {2\pi } \right)} \right) - n\left( {\log \left( {\alpha_{N} } \right) + \frac{1}{2}\log \left( {\beta_{N} } \right)} \right) \\ & \quad - \frac{3 n}{2} \mathop \sum \limits_{i = 1}^{n} Log\left( {x_{Ni} } \right) + \mathop \sum \limits_{i = 1}^{n} \log \left( {1 + \beta_{N} x_{Ni} } \right) - \frac{1}{{2 \alpha_{N}^{2} \beta_{N} }} \mathop \sum \limits_{i = 1}^{n} \frac{{\left( {\beta_{N} x_{Ni} - 1} \right)^{2} }}{{x_{Ni} }}. \\ \end{aligned} $$

Now get the derivatives of log-likelihood function with respect to ${\alpha }_{N}$ and ${\beta }_{N}$ to get the maximum likelihood estimates for ${\alpha }_{N}$ and ${\beta }_{N}$ which denoted by ${\widehat{\alpha }}_{N}$ and ${\widehat{\beta }}_{N}$ as follows:

$$\frac{\partial logL\left({x}_{Ni};{\alpha }_{N},{\beta }_{N}\right)}{\partial {\alpha }_{N}}=-\frac{n}{{\alpha }_{N}}+\frac{1}{{{\alpha }_{N}}^{3}{\beta }_{N}}\sum_{i=1}^{n}\frac{{\left(-1+{\beta }_{N}{x}_{Ni}\right)}^{2}}{{x}_{Ni}},$$

$$ \frac{{\partial logL\left( {x_{Ni} ;\alpha_{N} ,\beta_{N} } \right)}}{{\partial \beta_{N} }} = - \frac{n}{{2\beta_{N} }} + \frac{{\mathop \sum \nolimits_{i = 1}^{n} \frac{{\left( { - 1 + \beta_{N} x_{Ni} } \right)^{2} }}{{x_{Ni} }}}}{{2\alpha_{N}^{2} \beta_{N}^{2} }} + \mathop \sum \limits_{i = 1}^{n} \frac{{x_{Ni} }}{{1 + \beta_{N} x_{Ni} }} - \frac{{\mathop \sum \nolimits_{i = 1}^{n} \frac{{ - 2x_{Ni} + 2\beta_{N} x_{Ni}^{2} }}{{x_{Ni} }}}}{{2\alpha_{N}^{2} \beta_{N} }}. $$

Normal equations can’t solve analytic. So, we can’t get the closed form for ${\widehat{\alpha }}_{N}$ and ${\widehat{\beta }}_{N}$. Hence, numerical method is used to solve these equations. Since the maximum likelihood estimates for unknown parameters of new proposed distribution ${\widehat{\alpha }}_{N}$ and ${\widehat{\beta }}_{N}$ can’t get in closed form, so the exact distributions of these parameters not derived, so we derive the asymptotic confidence intervals of these parameters. For large sample and ${\alpha }_{N}>0$ and ${\beta }_{N}>0$. The ${\widehat{\alpha }}_{N}$ and ${\widehat{\beta }}_{N}$ are bivariate normal distribution with the mean ${\alpha }_{N}$ and ${\beta }_{N}$ and covariance matrix ${I}_{n}^{-1}$. Where ${I}_{n}^{-1}$ is the inverse of information matrix, where,

$${I}_{n}^{-1}=\left(\begin{array}{cc}Var({\widehat{\alpha }}_{N})& Cov({\widehat{\alpha }}_{N},{\widehat{\beta }}_{N})\\ Cov({\widehat{\alpha }}_{N},{\widehat{\beta }}_{N})& Var({\widehat{\beta }}_{N})\end{array}\right),$$

For more details see^57^. Now the $100\left(1-\gamma \right)\%$ confidence interval of parameters ${\alpha }_{N}$ and ${\beta }_{N}$ are ${\widehat{\alpha }}_{N}\pm {z}_{\gamma /2}\sqrt{Var({\widehat{\alpha }}_{N})}$ and ${\widehat{\beta }}_{N}\pm {z}_{\gamma /2}\sqrt{Var({\widehat{\beta }}_{N})}$ respectively.

### Bayesian estimation method

The Bayes estimation using MCMC technique is used to estimate the unknown parameters of new proposed distribution $NBS({\alpha }_{N},{\beta }_{N})$. For more details of MCMC technique using Gibb sampling procedure see^58,59^. Also, for more details of MCMC technique using Metropolis Hasting (MH) method see^60^ and^61^. The two methods are used to generate samples from the posterior density function to compute point Bayes estimators for unknown parameters and construct credible intervals. For this aim, we suppose that independent gamma prior distributions for unknown parameters of new proposed distribution $NBS({\alpha }_{N},{\beta }_{N})$ as follows:

$$ \pi_{1} \left( {\alpha_{N} } \right) = \frac{{b^{a} }}{{{\Gamma }\left( a \right)}} \alpha_{N}^{a - 1} {\text{exp}}\left( { - b \alpha_{N} } \right)\quad a > 0, b > 0, \alpha_{N} > 0, $$

$$ \pi_{2} \left( {\beta_{N} } \right) = \frac{{d^{c} }}{{{\Gamma }\left( c \right)}}{ }\beta_{N}^{c - 1} {\text{ exp}}\left( { - d{ }\beta_{N} } \right)\quad c > 0,{ }d > 0,{ }\beta_{N} > 0. $$

In this case the joint prior distribution of ${\alpha }_{N}$ and ${\beta }_{N}$ is given by,

$$\pi \left({\alpha }_{N},{\beta }_{N}\right)={\pi }_{1}\left({\alpha }_{N}\right){\pi }_{2}\left({\beta }_{N}\right),$$

And the joint posterior is given by,

$${\pi }^{*}\left({\alpha }_{N},{\beta }_{N}\left|{X}_{N}\right.\right)=\frac{L\left({\alpha }_{N},{\beta }_{N}\left|{X}_{N}\right.\right) \pi \left({\alpha }_{N},{\beta }_{N}\right)}{{\int }_{0}^{\infty }{\int }_{0}^{\infty }L\left({\alpha }_{N},{\beta }_{N}\left|{X}_{N}\right.\right) \pi \left({\alpha }_{N},{\beta }_{N}\right)d{\alpha }_{N}d{\beta }_{N}}.$$

Under square error loss the Bayes estimates of ${\alpha }_{N}$ and ${\beta }_{N}$ are given by.

$${\alpha }_{N}^{B}={\int }_{0}^{\infty }{\int }_{0}^{\infty }{\alpha }_{N} {\pi }^{*}\left({\alpha }_{N},{\beta }_{N}\left|{X}_{N}\right.\right) d{\beta }_{N}d{\alpha }_{N},$$

$${\beta }_{N}^{B}={\int }_{0}^{\infty }{\int }_{0}^{\infty }{\beta }_{N} {\pi }^{*}\left({\alpha }_{N},{\beta }_{N}\left|{X}_{N}\right.\right) d{\alpha }_{N}d{\beta }_{N}.$$

These estimates can’t be computed analytically. So, we use MCMC method using MH technique to get the ${\alpha }_{N}^{B}$ and ${\beta }_{N}^{B}$ as follows:

i. Choose initial values of ${\alpha }_{N}^{(0)}$ and ${\beta }_{N}^{(0)}.$
ii. Suppose the values of ${\alpha }_{N}$ and ${\beta }_{N}$ at the k^th^ step by ${\alpha }_{N}^{(k)}$ and ${\beta }_{N}^{(k)}.$
iii. Generate ${\alpha }_{N}^{(k)}$ using ${\pi }^{*}\left({\alpha }_{N}\left|{\beta }_{N}^{(k-1)},{X}_{N}\right.\right)$ and ${\pi }^{*}\left({\beta }_{N}\left|{{\alpha }_{N}^{(k-1)},X}_{N}\right.\right)$ respectively.
iv. Repeat step 3 N-times.
v. Compute Bayes estimates of ${\alpha }_{N}$ and ${\beta }_{N}$ as follows:
  $$ \alpha_{N}^{B} = \frac{1}{N - B} \mathop \sum \limits_{k = B + 1}^{N} \alpha_{N}^{\left( k \right)} ,\beta_{N}^{B} = \frac{1}{N - B} \mathop \sum \limits_{k = B + 1}^{N} \beta_{N}^{\left( k \right)} , $$
  where $B$ is the burn-in period.
vi Compute $\left(100-\gamma \right)\%$ HPD credible intervals for ${\alpha }_{N}$ and ${\beta }_{N}$ as follows:
  $$ \left( {\alpha_{{N\left( {\frac{\gamma }{2}} \right)}} ,\alpha_{{N\left( {1 - \frac{\gamma }{2}} \right)}} } \right),\left( {\beta_{{N\left( {\frac{\gamma }{2}} \right)}} ,\beta_{{N\left( {1 - \frac{\gamma }{2}} \right)}} } \right). $$

Note that we use R-Studio Software to get results in this section using many packages such that, nlme, MASS, coda, mcmc, distr, VGAM and RCPP.

## Simulation and comparative study

In this section, we perform Monte-Carlo simulation study to investigate the behavior of two different estimators for parameters of new proposed distribution $NBS({\alpha }_{N},{\beta }_{N})$ maximum likelihood estimators and Bayesian estimates according to different sample sizes, different start values of ${\alpha }_{N}$ and ${\beta }_{N}$ and different indeterminacy measure. Also, we introduce comparative study to compare between maximum likelihood estimates (MLE’s) and Bayesian estimates to get the best for parameters of new proposed distribution $NBS({\alpha }_{N},{\beta }_{N})$. Compare between classical and neutrosophic version of BS distribution to show the flexibility of neutrosophic version. Finally, compare Bayesian estimates for different prior distributions. For the aim of comparative study, we use bias and mean square error (MSE) to compare between different point estimators. Use also the Akaike information criterion (AIC) to compare maximum likelihood estimator for classical and neutrosophic version but use asymptotic confidence length (ACL) to compare between different interval estimators. Now we perform these studies according to the following steps:

i. Choose the different initial values of $\left({\alpha }_{N},{\beta }_{N}\right)=\left(\mathrm{1.25,3}\right), \left(\mathrm{0.5,3}\right), \left(\mathrm{1,3}\right).$
ii. For Bayesian estimators choose different values of the parameter for gamma prior follows $\left(a,b\right)=\left(\mathrm{1,2}\right), \left(\mathrm{1,1}\right), (\mathrm{2,1})$.
iii. Use two indeterminacy measure ${I}_{N}=\left(\mathrm{0.2,0.5}\right), (\mathrm{0.6,0.8})$. Note that when ${I}_{N}=0$, the classical version of Birnbaum Saunders is obtained.
iv. Generate different sample sizes $n=50, 100, 200, 500.$
v. Find point estimators using maximum likelihood estimation method and Bayesian estimation method.
vi. Calculate asymptotic confidence interval and credible interval.
vii. Perform the comparative study by calculating bias, MSE and AIC to compare MLE’s in both cases classical and neutrosophic version of BS distribution, calculating bias, MSE for comparing MLE’s and Bayesian estimates in both cases classical and neutrosophic version of BS distribution. And use ACL for comparing interval estimation.

All calculations in this section we use R package For more details about R-Package see^62^. Results of simulation and comparative study between our new proposed distribution neutrosophic Birnbaum–Saunders distribution and its classical version Birnbaum–Saunders distribution are shown in Tables 1, 2, 3, 4, 5 and 6 which get using R-Studio Software. From These Tables, we get, Tables 1 and 2 shown bias’s, MSE’s and AIC for MLE’s for ${\text{BS}}(\mathrm{\alpha },\upbeta )$ and ${\text{NBS}}({\mathrm{\alpha }}_{{\text{N}}},{\upbeta }_{{\text{N}}})$ respectively, we get ${\text{NBS}}({\mathrm{\alpha }}_{{\text{N}}},{\upbeta }_{{\text{N}}})$ has smaller bias’s, MSE’s and AIC than ${\text{BS}}\left(\mathrm{\alpha },\upbeta \right)$ so, we can decided that the neutrosophic version is better than the classical version. Also, in the context of interval estimation the ${\text{NBS}}({\mathrm{\alpha }}_{{\text{N}}},{\upbeta }_{{\text{N}}})$ has smaller ACL for asymptotic confidence interval than ${\text{BS}}(\mathrm{\alpha },\upbeta )$. So, For MLE’s the ${\text{NBS}}({\mathrm{\alpha }}_{{\text{N}}},{\upbeta }_{{\text{N}}})$ has good behavior than ${\text{BS}}\left(\mathrm{\alpha },\upbeta \right).$ Also, we get for two versions bias’s and MSE’s decrease when sample size increase. For Bayesian estimator results are shown in Tables 3, 4, 5 and 6, we get also, ${\text{NBS}}({\mathrm{\alpha }}_{{\text{N}}},{\upbeta }_{{\text{N}}})$ has smaller bias’s and MSE’s than ${\text{BS}}\left(\mathrm{\alpha },\upbeta \right)$ as shown in Tables 5 and 6. Also, for credible intervals ${\text{NBS}}({\mathrm{\alpha }}_{{\text{N}}},{\upbeta }_{{\text{N}}})$ has smaller ACL than ${\text{BS}}\left(\mathrm{\alpha },\upbeta \right).$ The behavior of Bayesian estimation got for different three prior distributions. So, we can decide also, ${\text{NBS}}({\mathrm{\alpha }}_{{\text{N}}},{\upbeta }_{{\text{N}}})$ has good behavior than ${\text{BS}}\left(\mathrm{\alpha },\upbeta \right)$.

**Table 1:** Bias’s, MSE’s, AIC’s and ACL’s for MLE’s for $BS\left(\alpha ,\beta \right).$

n	$BS(\alpha ,\beta )$						
	$\alpha$		$\beta$		AIC	$\alpha$	$\beta$
	Bias	MSE	Bias	MSE		ACL	
$$\left(\alpha ,\beta \right)=\left(\mathrm{1.25.5,3}\right)$$							
50	0.0193	0.0188	0.03350	0.0563	9963.393	0.1654	0.5138
100	0.0088	0.0077	0.0165	0.0273	32,422.27	0.1563	0.3786
200	0.0045	0.0041	0.0084	0.0141	132,107.5	0.0998	0.2654
500	0.0018	0.0016	0.0034	0.0060	826,131.3	0.0593	0.1550
$$\left(\alpha ,\beta \right)=\left(\mathrm{0.5,3}\right)$$							
50	0.0039	0.0007	0.0508	0.1292	8980.385	0.0744	0.1795
100	0.0018	0.0003	0.0239	0.0572	34,746.18	0.0716	0.0167
200	0.0007	0.0001	0.0125	0.0314	128,830.6	0.0453	0.0960
500	0.0003	5.16*${10}^{-5}$	0.0050	0.0125	810,005.7	0.0283	0.0618
$$\left(\alpha ,\beta \right)=\left(\mathrm{1,3}\right)$$							
50	0.0138	0.0095	0.0428	0.0916	8744.334	0.1340	0.3390
100	0.0069	0.0048	0.0204	0.0419	36,114.63	0.1169	0.2579
200	0.0034	00,023	0.0102	0.0208	139,516.5	0.0743	0.1876
500	0.0013	0.0008	0.0040	0.0080	812,134.2	0.0516	0.1246

**Tablae 2:** Bias’s, MSE’s, AIC’s and ACL’s for MLE’s for $NBS(\alpha ,\beta )$.

n	$NBS({\alpha }_{N},{\beta }_{N})$						
	${\alpha }_{N}$		${\beta }_{N}$		AIC	${\alpha }_{N}$	${\beta }_{N}$
	Bias	MSE	Bias	MSE		ACL	
$\left({\alpha }_{N},{\beta }_{N}\right)=\left(\mathrm{1.25,3}\right)$, ${I}_{N}=\left(\mathrm{0.2,0.5}\right)$							
50	(0.0093, 0.0153)	(0.0044, 0.0118)	(0.0235, 0.0295)	(0.0278, 0.0437)	(9922.846, 9945.161)	(0.1651, 0.1652)	(0.5118, 0.5128)
100	(0.0038, 0.0068)	(0.0014, 0.0046)	(0.0115, 0.0145)	(0.0133, 0.0211)	(32,341.18, 32,385.81)	(0.1556, 0.1562)	(0.3754, 0.3767)
200	(0.0020, 0.0035)	(0.0008, 0.0025)	(0.0059, 0.0074)	(0.0070, 0.0110)	(131,945.3, 132,034.5)	(0.0005, 0.0007)	(0.2492, 0.2653)
500	(0.0008, 0.0014)	(0.0003, 0.0010)	(0.0024, 0.0030)	(0.0030, 0.0047)	(825,725.8, 825,949)	(0.0587, 0.0590)	(0.14831, 0.1524)
$\left({\alpha }_{N},{\beta }_{N}\right)=\left(\mathrm{0.5,3}\right)$, ${I}_{N}=\left(\mathrm{0.2,0.5}\right)$							
50	(− 0.0060 $$,$$ − 2.8*$${10}^{-5})$$	(4.09*$${10}^{-8}$$, 0.0006 $$)$$	(0.0408, 0.0468)	(0.0834, 0.1097)	(8939.838, 8962.153)	(0.0740, 0.0743)	(0.1782, 0.1792)
100	(− 0.0003, − 0.0001)	(2.6*${10}^{-6}$, 0.0002)	(0.0189, 0.0219)	(0.0358, 0.0481)	(34,665.09, 34,709.72)	(0.0710, 0.0713)	(0.0162, 0.0163)
200	(− 0.0002, − 0.0017)	(9.01*${10}^{-6},$ 0.0001)	(0.0100, 0.0115)	(0.0201, 0.0266)	(128,668.4, 128,757.6)	(0.0450, 0.0452)	(0.0952, 0.0958)
500	(− 7.8*${10}^{-5},$ − 0.0006)	(3.08*${10}^{-6},$ 0.0001)	(0.0040, 0.0046)	(0.0080, 0.0106)	(809,600.2, 809,823.4)	(0.0280, 0.0282)	(0.0613, 0.0614)
$\left({\alpha }_{N},{\beta }_{N}\right)=\left(\mathrm{1,3}\right)$, ${I}_{N}=\left(\mathrm{0.2,0.5}\right)$							
50	(0.0038, 0.0098)	(0.0007, 0.0048)	(0.0328, 0.0388)	(0.0538, 0.07534)	(8703.788, 8726.102)	(0.1338, 0.1339)	(0.3367, 0.3378)
100	(0.0019, 0.0049)	(0.0003, 0.0024)	(0.0154, 0.0184)	(0.0239, 0.0341)	(36,033.54, 36,078.17)	(0.1156, 0.01159)	(0.2558, 0.2569)
200	(0.0009, 0.0024)	(0.0001, 0.0011)	(0.0077, 0.0062)	(0.0118, 0.0169)	(139,354.3, 139,443.5)	(0.0740, 0.0742)	(0.1816, 0.1845)
500	(0.0009, 0.0003)	(5.45*${10}^{-5}$, 0.0004)	(0.0030, 0.0036)	(0.0045, 0.0065)	(811,728.7, 811,951.8)	(0.0512, 0.0514)	(0.1241, 0.1245)
$\left({\alpha }_{N},{\beta }_{N}\right)=\left(\mathrm{1.25,3}\right)$, ${I}_{N}=\left(\mathrm{0.6,0.8}\right)$							
50	(− 0.0001, 0.0038)	(8.32*${10}^{-9},$ 0.0007)	(0.0154, 0.0190)	(0.0120, 0.0190)	(5667.594, 5679.373)	(0.0819, 0.0823)	(0.4594, 0.4646)
100	(0.0009, 0.0029)	(8.46*${10}^{-5},$ 0.0008)	(0.0101, 0.0121)	(0.0103, 0.0148)	(30,906.83, 30,930.38)	(0.0395, 0.0396)	(0.3249, 0.3290)
200	(0.0005, 0.0006)	(5.15*${10}^{-5},$ 0.0004)	(0.0044, 0.0054)	(0.0040, 0.0060)	(122,478.9, 122,526)	(0.0017, 0.0022)	(0.2528, 0.2529)
500	(0.0002, 0.0006)	(2.84*${10}^{-5},$ 0.0002)	(0.0018, 0.0022)	(0.0017, 0.0025)	(844,985.4, 845,573.2)	(0.0401, 0.0410)	(0.1425, 0.1513)
$\left({\alpha }_{N},{\beta }_{N}\right)=\left(\mathrm{0.5,3}\right)$, ${I}_{N}=\left(\mathrm{0.6,0.8}\right)$							
50	(− 1.0131, − 0.0091)	(0.0041, 0.0086)	(0.0341, 0.0381)	(0.0584, 0.0729)	(7632.389, 7644.168)	(0.0640, 0.0641)	(0.1620, 0.1622)
100	(− 0.0063, − 0.0043)	(0.0019, 0.0040)	(0.0168, 0.0188)	(0.0284, 0.0355)	(32,526.3, 32,549.85)	(0.0656, 0.0658)	(0.1423, 0.1426)
200	(− 0.0031, − 0.0021)	(0.0009, 0.0019)	(0.0082, 0.0092)	(0.0137, 0.0172)	(134,481.5, 134,528.6)	(0.0422, 0.0433)	(0.0051, 0.0054)
500	(− 0.0012, − 0.0008)	(0.0003, 0.0007)	(0.0033, 0.0037)	(0.0057, 0.0071)	(840,294.8, 840,412.6)	(0.0281, 0.0282)	(0.0523, 0.0532)
$\left({\alpha }_{N},{\beta }_{N}\right)=\left(\mathrm{1,3}\right)$, ${I}_{N}=\left(\mathrm{0.6,0.8}\right)$							
50	(− 0.0025, 0.0014)	(0.0001, 0.0033)	(0.0255, 0.0295)	(0.0325, 0.0435)	(8349.03, 8360.808)	(0.1208, 0.1209)	(0.3618, 0.3647)
100	(− 0.0019, 6.38*$${10}^{-5})$$	(4.07*$${10}^{-7},$$ 0.0003)	(0.0126, 0.0146)	(0.0159, 0.0214)	(27,309.47, 27,333.03)	(0.1107, 0.1109)	(0.2479, 0.2499)
200	(− 0.0007, 0.0002)	(1.60*${10}^{-5},$ 0.0001)	(0.0060, 0.0070)	(0.0073, 0.0099	(129,253.6, 129,300.7)	(0.0630, 0.0632)	(0.1808, 0.1824)
500	(− 0.0002, 0.0001)	(6.08*$${10}^{-6},$$ 4.19*$${10}^{-5})$$	(0.0024, 0.0028)	(0.0030, 0.0041)	(800,043, 800,160.8)	(0.0513, 0.0515)	(0.1178, 0.1244)

**Table 3:** The ALC’s for Credible intervals for $BS\left(\alpha ,\beta \right).$

n	Credible intervals					
	$$BS(\alpha ,\beta )$$					
	$$\left(\alpha ,\beta \right)=\left(\mathrm{1.25,3}\right)$$					
	ACL					
	Prior 1		Prior 2		Prior3	
	$\alpha$	$\beta$	$\alpha$	$\beta$	$\alpha$	$\beta$
50	0.9457	0.9439	0.9533	0.9322	0.9385	0.9589
100	0.9411	0.9545	0.9462	0.9315	0.9471	0.9604
200	0.9560	0.9337	0.9608	0.9458	0.9594	0.9474
500	0.9522	0.9245	0.9362	0.9513	0.9549	0.9313
$$\left(\alpha ,\beta \right)=\left(\mathrm{0.5,3}\right)$$						
50	0.9406	0.9413	0.9480	0.9517	0.9443	0.9607
100	0.9448	0.9422	0.9296	0.9505	0.9416	0.9395
200	0.9429	0.9313	0.9281	0.9530	0.9251	0.9441
500	0.9466	0.9486	0.9489	0.9465	0.9353	0.9521
$$\left(\alpha ,\beta \right)=\left(\mathrm{1,3}\right)$$						
50	0.9279	0.9498	0.9489	0.9378	0.9442	0.9456
100	0.9531	0.9385	0.9372	0.9425	0.9439	0.9499
200	0.9330	0.9340	0.9389	0.9498	0.9382	0.9372
500	0.9423	0.9378	0.9411	0.9414	0.9513	0.9437

**Table 4:** The ALC’s for credible intervals for $NBS\left(\alpha ,\beta \right).$

n	Credible intervals					
	$$NBS(\alpha ,\beta )$$					
	$\left({\alpha }_{N},{\beta }_{N}\right)=\left(\mathrm{1.25,3}\right)$, ${I}_{N}=\left(\mathrm{0.2,0.5}\right)$					
	ACL					
	Prior 1		Prior 2		Prior3	
	${\alpha }_{N}$	${\beta }_{N}$	${\alpha }_{N}$	${\beta }_{N}$	${\alpha }_{N}$	${\beta }_{N}$
50	(0.9299, 0.9450)	(0.9406, 0.9420)	(0.9321, 0.9329)	(0.9276, 0.9238)	(0.9286, 0.9295)	(0.9466, 0.9538)
100	(0.9389, 0.9391)	(0.9380, 0.9466)	(0.9435, 0.9446)	(0.9257, 0.9280)	(0.9465, 0.9473)	(0.9491, 0.9578)
200	(0.9362, 0.9387)	(0.9227, 0.9324)	(0.9513, 0.9542)	(0.9389, 0.9401)	(0.9584, 0.9586)	(0.9213, 0.9456)
500	(0.9402, 0.9419)	(0.9212, 0.9222)	(0.9391, 0.9579)	(0.9271, 0.9403)	(0.9464, 0.9540)	(0.9240, 0.9262)
$\left({\alpha }_{N},{\beta }_{N}\right)=\left(\mathrm{0.5,3}\right)$, ${I}_{N}=\left(\mathrm{0.2,0.5}\right)$						
50	(0.9322, 0.9518)	(0.9386, 0.9393)	(0.9433, 0.9478)	(0.9433, 0.9487)	(0.9400, 0.9441)	(0.9416, 0.9492)
100	(0.9296, 0.9317)	(0.9338, 0.9344)	(0.9246, 0.9267)	(0.9339, 0.9468)	(0.9362, 0.9415)	(0.9353, 0.9392)
200	(0.9192 0.9337)	(0.9228, 0.9234)	(0.9195, 0.9267)	(0.9446, 0.9461)	(0.9194, 0.9212)	(0.9402, 0.9520)
500	(0.9402, 0.9417)	(0.9333, 0.9458)	(0.9333, 0.9437)	(0.9378, 0.9451)	(0.9307, 0.9341)	(0.9426, 0.9433)
$\left({\alpha }_{N},{\beta }_{N}\right)=\left(\mathrm{1,3}\right)$, ${I}_{N}=\left(\mathrm{0.2,0.5}\right)$						
50	(0.9146, 0.9203)	(0.9354, 0.9362)	(0.9471, 0.9485)	(0.9382, 0.9433)	(0.9323, .9335)	(0.9348, 0.9500)
100	(0.9432, 0.9434)	(0.9339 0.9384)	(0.9363, 0.9364)	(0.9315, 0.9436)	(0.9247, 0.9406)	(0.9371, .9390)
200	(0.9287, 0.9318)	(0.9293, 0.9301)	(0.9337, 0.9349)	(0.9377, 0.9404)	(0.9125, 0.9232)	(0.9362, 0.9349)
500	(0.9349, 0.9394)	(0.9312, 0.9374)	(0.9262, 0.9320)	(0.9430, 0.9451)	(0.9313, 0.9340)	(0.9304, 0.9366)
$\left({\alpha }_{N},{\beta }_{N}\right)=\left(\mathrm{1.25,3}\right)$, ${I}_{N}=\left(\mathrm{0.6,0.8}\right)$						
50	(0.9443 0.9446)	(0.9301, 0.9409)	(0.9355, 0.9488)	(0.9285, 0.9293)	(0.9250, 0.9295)	(0.9457, 0.9576)
100	(0.9250, 0.9316)	(0.9391, 0.9446)	(0.9356, 0.9423)	(0.9289, 0.9298)	(0.9407, 0.9462)	(0.9351, 0.9597)
200	(0.9351, 0.9434)	(0.9265, 0.9274)	(0.9368, 0.9492)	(0.9443, 0.9457)	(0.9270, 0.9319)	(0.9314, 0.9409)
500	(0.9382, 0.9439)	(0.9175, 0.9201)	(0.9325, 0.9344)	(0.9458, 0.9462)	(0.9418, 0.9434)	(0.9232, 0.9274)
$\left({\alpha }_{N},{\beta }_{N}\right)=\left(\mathrm{0.5,3}\right)$, ${I}_{N}=\left(\mathrm{0.6,0.8}\right)$						
50	(0.9314, 0.9323)	(0.9335, 0.9373)	(0.9249, 0.9380)	(0.9458, 0.9461)	(0.9370, 0.9378)	(0.9369, 0.9605)
100	(0.9342, 0.9444)	(0.9416, 0.9420)	(0.9137, 0.9215)	(0.9470, 0.9482)	(0.9360, 0.9377)	(0.9344, 0.9394)
200	(0.9309, 0.9318)	(0.9300, 0.9302)	(0.9162, 0.9204)	(0.9442, 0.9448)	(0.9163, 0.9222)	(0.9416, 0.9433)
500	(0.9455, 0.9464)	(0.9471, 0.9484)	(2.9324, 2.9396)	(0.9245, 0.9375)	(0.9348, 0.9350)	(0.9394, 0.9443)
$\left({\alpha }_{N},{\beta }_{N}\right)=\left(\mathrm{1,3}\right)$, ${I}_{N}=\left(\mathrm{0.6,0.8}\right)$						
50	(0.9220, 0.9259)	(0.9456, 0.9464)	(0.9318, 0.9420)	(0.9238, 0.9366)	(0.9370, 0.9389)	(0.9381, 0.9433)
100	(0.9523, 0.9524)	(0.9323, 0.9341)	(0.9178, 0.9249)	(0.9338, 0.9411)	(0.9287, 0.9410)	(0.9452, 0.9460)
200	(0.9311, 0.9321)	(0.9314, 0.9321)	(0.9334, 0.9373)	(0.9373, 0.9446)	(0.9272, 0.9362)	(0.9328, 0.9352)
500	(0.9383, 0.9420)	(0.9356, 0.9366)	(0.9335, 0.9347)	(0.9305, 0.9405)	(0.9381, 0.9465)	(0.9403, 0.9429)

**Table 5:** Bias’s and MSE’s for Bayesian estimation for $BS(\alpha ,\beta )$.

n	$BS(\alpha ,\beta )$											
	Prior 1				Prior 2				Prior3			
	$\alpha$		$\beta$		$\alpha$		$\beta$		$\alpha$		$\beta$	
	Bias	MSE	Bias	MSE	Bias	MSE	Bias	MSE	Bias	MSE	Bias	MSE
$$\left(\alpha ,\beta \right)=\left(\mathrm{1.25,3}\right)$$												
50	0.0119	0.0075	0.0175	0.0306	0.0144	0.0131	0.0172	0.0316	0.0076	0.0062	0.0135	0.0106
100	0.0096	0.0446	0.0087	0.0153	0.0080	0.0314	0.0085	0.0163	0.0036	0.0013	0.0088	0.0123
200	0.0032	0.0030	0.0043	0.0076	0.0042	0.0209	0.0041	0.0066	0.0015	0.0010	0.0013	0.0036
500	0.0058	0.0366	0.0017	0.0030	0.0014	0.0011	0.0015	0.0027	0.0021	0.0022	0.0014	0.0003
$$\left(\alpha ,\beta \right)=\left(\mathrm{0.5,3}\right)$$												
50	0.0084	0.0062	0.0175	0.0306	− 0.0008	0.0018	0.0250	0.0625	− 0.0040	0.0064	0.0030	0.0070
100	0.0086	0.0221	0.0087	0.0153	0.0035	0.0013	0.0125	0.0312	− 0.0050	0.0100	0.0050	0.0212
200	0.0041	0.0080	0.0043	0.0076	0.0011	0.0005	0.0062	0.0156	7.8 $*{10}^{-7}$	0.0002	0.0033	0.0125
500	0.0025	0.0081	0.0017	0.0030	− 0.0011	0.0008	0.0025	0.0006	− 0.0013	0.0037	0.0012	0.0003
$$\left(\alpha ,\beta \right)=\left(\mathrm{1,3}\right)$$												
50	0.0135	0.0109	0.0200	0.0400	0.0046	0.0094	0.0019	0.0038	0.0027	0.0073	0.0150	0.0425
100	0.0030	0.0007	0.0100	0.0200	0.0028	0.0010	0.0009	0.0019	0.0051	0.0049	0.0075	0.0212
200	0.0024	0.0012	0.0050	0.0100	0.0041	0.0034	0.0048	0.0018	− 0.0007	0.0002	0.0038	0.0106
500	0.0016	0.0014	0.0020	0.0040	0.0009	0.0006	0.0018	0.0038	− 0.0041	0.0187	0.0015	0.0043

**Table 6:** Bias’s and MSE’s for Bayesian estimation for $NBS(\alpha ,\beta )$.

n	$NBS(\alpha ,\beta )$											
	Prior 1				Prior 2				Prior3			
	${\alpha }_{N}$		${\beta }_{N}$		${\alpha }_{N}$		${\beta }_{N}$		${\alpha }_{N}$		${\beta }_{N}$	
	Bias	MSE	Bias	MSE	Bias	MSE	Bias	MSE	Bias	MSE	Bias	MSE
$\left({\alpha }_{N},{\beta }_{N}\right)=\left(\mathrm{1.25,3}\right)$, ${I}_{N}=\left(\mathrm{0.2,0.5}\right)$												
50	(0.0064, 0.0088)	(0.0023, 0.0039)	(0.0075 0.0115)	(0.0180, 0.0244)	(0.0041, 0.0134)	(0.0018, 0.0129)	(0.0065, 0.0135)	(0.0181, 0.0243)	(− 0.0069, − 0.0063)	(0.0034, 0.0100)	(0.0126, 0.0130)	(0.0006, 0.0009)
100	(0.0014, 0.0021)	(0.0011, 0.0031)	(0.0038, 0.0067)	(0.0091, 0.0122)	(0.0037, 0.0047)	(0.0014, 0.0022)	(0.0037, 0.0068)	(0.0090, 0.0122)	(0.0091, 0.0015)	(0.0007, 0.0012)	(0.0039, 0.0086)	(0.0113, 0.0121)
200	(− 0.0169, − 0.0028)	(0.0024, 0.0029)	(0.0019. 0.0031)	(0.0045, 0.0061)	(− 0.0016, 0.0016)	(0.0006, 0.0052)	(0.0018, 0.0034)	(0.0043, 0.0062)	(0.0011, 0.0022)	(0.0004, 0.0009)	(0.0002, 0.0009)	(0.0004, 0.0006)
500	(0.0001, 0.0006)	(0.0005, 0.0017)	(0.0008, 0.0014)	(0.0018, 0.0024)	(0.008, 0.0009)	(0.0003, 0.0004)	(0.0007, 00,013)	(0.0019, 0.0025)	(− 0.0007, 0.0020)	(1.6*${10}^{-5}$ 0.0012)	(0.0012, 0.0013)	(0.0001, 0.0003)
$\left({\alpha }_{N},{\beta }_{N}\right)=\left(\mathrm{0.5,3}\right)$, ${I}_{N}=\left(\mathrm{0.2,0.5}\right)$												
50	(− 0.0334 − 0.0016)	(0.0003, 0.0004)	(0.0150, 0.0170)	(0.0300, 0.0303)	(− 0.0356, − 0.0084)	(0.0009, 0.0015)	(0.0020, 0.0250)	(0.0522 0.0625)	(− 0.0139, − 0.0133)	(0.0091, 0.0043)	(0.0025, 0.0330)	(0.0062, 0.0067)
100	(− 0.0093, − 0.0063)	(0.0064, 0.0096)	(0.0075, 0.0085)	(0.0113, 0.0127)	(− 0.0103, − 0.0102)	(0.0010, 0.0012)	(0.0115, 0.0117)	(0.0212, 0.0302)	(− 0.0177, − 0.0004)	(0.0006, 0.0009)	(0.0025, 0.0035)	(0.0112, 0.0147)
200	(− 0.0099, − 0.0073)	(0.0040, 0.0074)	(0.0038, 0.0042)	(0.0066, 0.0073)	(− 0.0046, − 0.0101)	(0.0001, 0.0004)	(0.0053, 0.0057)	(0.0106, 0.0146)	(− 0.0114, 0.0013)	(0.0001, 0.0002)	(0.0013, 0.0025)	(0.0100, 0.0114)
500	(− 0.0023, − 0.0011)	(0.0008, 0.0027)	(0.0012, 0.0016)	(0.0025, 0.0030)	(− 0.0011, − 0.0005)	(0.0002, 0.0007)	(0.0015, 0.0023)	(0.0002, 0.0006)	(− 0.0028, − 0.0021)	(0.0033, 0.0036)	(0.0006, 0.0010)	(0.0001, 0.0002)
$\left({\alpha }_{N},{\beta }_{N}\right)=\left(\mathrm{1,3}\right)$, ${I}_{N}=\left(\mathrm{0.2,0.5}\right)$												
50	(9.40*${10}^{-5},$ 0.0081)	(0.0024, 0.0054)	(0.0100, 0.0160)	(0.0250, 0.0328)	(0.0023, 0.0039)	(0.0004, 0.0018)	(0.0019, 0.0040)	(0.0009, 0.0018)	(− 0.0418, − 0.0329)	(0.0022, 0.0026)	(0.0001, 0.00130)	(0.0200, 0.0400)
100	(− 0.0048, − 0.0026)	(0.0001, 0.0007)	(0.0050, 0.0080)	(0.0125, 0.0164)	(− 0.0038, − 0.0036)	(0.0015, 0.0021)	(0.0009, 0.0010)	(0.0002, 0.0005)	(− 0.0020, 0.0004)	(0.0009, 0.0013)	(0.0008, 0.0060)	(0.0100, 0.0200)
200	(− 0.0042, − 0.0002)	(0.0006, 0.0011)	(0.0025, 0.0040)	(0.0065, 0.0082)	(5.49*${10}^{-5}$, 0.0001)	(0.0002, 0.0031)	(0.0025, 0.0030)	(0.0030, 0.0035)	(− 0.0034, − 0.0100)	(0.0001, 0.0002)	(0.0004, 0.0020)	(0.0050, 0.0100)
500	(− 0.0021, − 0.0004)	(0.0005, 0.0012)	(0.0010, 0.0016)	(0.0025, 0.0033)	(− 0.0026, 4.82*$${10}^{-5})$$	(5.6*$${10}^{-6},$$ 0.0099)	(0.0001, 0.0002)	(0.0010, 0.0015)	(− 0.0023, − 0.0008)	(0.0006, 0.0034)	(0.0005, 0.0019)	(0.0031, 0.0040)
$\left({\alpha }_{N},{\beta }_{N}\right)=\left(\mathrm{1.25,3}\right)$, ${I}_{N}=\left(\mathrm{0.6,0.8}\right)$												
50	(− 0.0116, − 0.0026)	(0.0010, 0.0028)	(0.0012, 0.0055)	(0.0154, 0.0168)	(− 0.0293, − 0.0010)	(0.0060, 0.0067)	(0.0145, 0.0165)	(0.0306, 0.0308)	(− 0.0026, 0.0028)	(0.0041, 0.0045)	(0.0125, 0.0130)	(0.0096, 0.0104)
100	(− 0.0236, 0.0003)	(0.0033, 0.0086)	(0.0008, 0.0028)	(0.0077, 0.0084)	(− 0.0171, 0.0032)	(0.0011, 0.0254)	(0.0067, 0.0078)	(0.0153, 0.0159)	(− 0.0051, 0.0032)	(0.0007, 0.0013)	(0.0080, 0.0087)	(0.0114, 0.0117)
200	(− 0.0041, 0.0029)	(0.0017, 0.0026)	(0.0004, 0.0014)	(0.0038, 0.0042)	(− 0.0092, − 0.0059)	(0.0113, 0.0157)	(0.0040, 0.0041)	(0.0056, 0.0064)	(− 0.0005, − 0.0036)	(6.2*${10}^{-5}$ 0.0004)	(0.0007, 0.0012)	(0.0027, 0.0033)
500	(− 0.0014, 0.0001)	(4.48*${10}^{-5},$ 0.0027)	(0.0001, 0.0006)	(0.0015, 0.0017)	(− 0.0021, 0.0012)	(0.0002, 0.0007)	(0.0008, 0.0013)	(0.0017, 0.0020)	(− 0.0031, 0.0008)	(0.0014, 0.0020)	(0.0010, 0.0013)	(0.0001, 0.0003)
$\left({\alpha }_{N},{\beta }_{N}\right)=\left(\mathrm{0.5,3}\right)$, ${I}_{N}=\left(\mathrm{0.6,0.8}\right)$												
50	(− 0.02901, − 0.0283)	(0.0056, 0.0059)	(0.0090, 0.0130)	(0.0253, 0.0297)	(− 0.0169, − 0.0105)	(0.0009, 0.0018)	(0.0150, 0.0170)	(0.0530, 0.0607)	(− 0.0218, 0.0122)	(0.0052, 0.0054)	(0.0020, 0.0025)	(0.0005, 0.0007)
100	(− 0.0093, − 0.0008)	(0.0083, 0.0088)	(0.0045, 0.0065)	(0.0126, 0.0148)	(− 0.0300, − 0.0089)	(0.0005, 0.0008)	(0.0110, 0.0125)	(0.0213, 0.0288)	(− 0.0074, 0.0003)	(0.0039, 0.0057)	(0.0021, 0.0035)	(0.0112, 0.0115)
200	(− 0.0148, − 0.0065)	(0.0076, 0.0080)	(0.0023, 0.0033)	(0.0058, 0.0069)	(− 0.0048, − 0.0021)	(0.0001, 0.0004)	(0.0022, 0.0042)	(0.0126, 0.0140)	(− 0.0127, 0.0014)	(0.0001, 0.0002)	(− 0.0017, 0.0022)	(0.0084, 0.0111)
500	(− 0.0034, − 0.0021)	(0.0027, 0.0067)	(0.0001, 0.0013)	(0.0025, 0.0029)	(− 0.0020, − 0.0009)	(0.0004, 0.0007)	(0.0015, 0.0025)	(0.0002, 0.0004)	(− 0.0013, − 0.0008)	(0.0003, 0.0009)	(− 4*${10}^{-4},$ 0.0011)	(0.0001, 0.0003)
$\left({\alpha }_{N},{\beta }_{N}\right)=\left(\mathrm{1,3}\right)$, ${I}_{N}=\left(\mathrm{0.6,0.8}\right)$												
50	(− 0.0262, − 0.0010)	(0.0007, 0.0108)	(0.0010, 0.0019)	(0.0232, 0.0208)	(− 0.0381, − 0.0124)	(0.0011, 0.0064)	(0.0014, 0.0019)	(0.0030, 0.0035)	(− 0.0401, 0.0058)	(0.0054, 0.0059)	(− 0.0120, 0.0019)	(0.0272, 0.0039)
100	(− 0.0063, − 0.0002)	(0.0006, 0.0007)	(0.0004, 0.0009)	(0.0116, 0.0104)	(− 0.0065, 0.0005)	(0.0003, 0.0009)	(0.0005, 0.0006)	(0.0012, 0.0017)	(− 0.0363, 0.0139)	(0.0033, 0.0044)	(− 0.0120, 0.0080)	(0.0018, 0.0173)
200	(− 0.0058, − 0.0055)	(0.0009, 0.0011)	(0.0010, 0.0020)	(0.0052, 0.0058)	(− 0.0033, − 0.0017)	(0.0019, 0.0025)	(0.0030, 0.0045)	(0.0010, 0.0018)	(− 0.0039, 0.0017)	(0.0001, 0.0002)	(− 0.003, 0.0050)	(0.0068, 0.0100)
500	(− 0.0043, − 0.0003)	(0.0003, 0.0013)	(0.0001, 0.0004)	(0.0021, 0.0023)	(− 0.0032, − 0.0007)	(0.0001, 0.0004)	(0.0012, 0.0018)	(0.0021, 0.0025)	(− 0.0029, 0.0002)	(0.0021, 0.0044)	(− 0.0012, 0.0020)	(0.0023, 0.0040)

## Comparative study using real application

The main aim of this section is a comparative study between the $NBS({\alpha }_{N},{\beta }_{N})$ and $BS\left(\alpha ,\beta \right)$. We introduced two real applications as follows:

### Application 1

Based on data of alloy melting points. For more details see^63^ which mentioned that A combination of material constituents, including at least one metal, makes up an alloy. In general, evaluating melting points is quite challenging, therefore observations are indeterministic and can be communicated in intervals as follows:

[563.3, 545.5], [529.4, 511.6], [523.1, 503.5], [470.1,449.2], [506.7, 489.0], [495.6, 479.1], [495.3, 467.9],[520.9, 495.6], [496.9, 472.8], [542.9, 519.1], [505.4,484.0], [550.7, 525.9], [517.7, 500.9], [499.2, 483.0],[500.6, 480.0], [516.8, 499.6], [535.0, 515.1], [489.3,464.4].

### Application 2

The data represent the lifetime of batteries. The lifetime in 100hours of 23 batteries is given as:

[2.9,3.99], [5.24,7.2],[6.56,9.02], [7.14,9.82], [11.6,15.96], [12.14,16.69], [12.65,17.4], [13.24,18.21], [13.67,18.79], [13.88,19.09], [15.64,21.51], [17.05,23.45], [17.4,23.93], [17.8,24.48], [19.01,26.14], [19.34,26.59], [23.13,31.81], [23.34,32.09],[26.07,35.84], [30.29,41.65], [43.97,60.46], [48.09,66.13], [73.48,98.04]. For more details see^64^^.^

**Figure 7:**
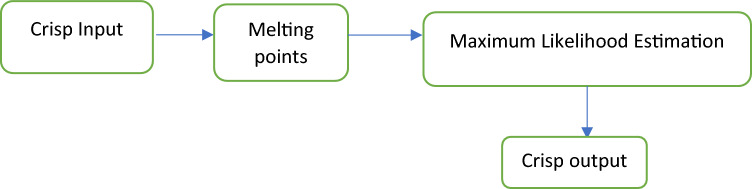
Architectural Diagram of the Proposed Algorithm.

Figure 7 shows the architectural diagram of the proposed algorithm in this section. To show the performance of our new distribution the $NBS({\alpha }_{N},{\beta }_{N})$. We compare it with its classical version $BS\left(\alpha ,\beta \right)$ using three statistical criteria log -likelihood (− 2LL), Akaike’s Information Criteria (AIC) and Bayesian Information Criteria (BIC), where,

$$ AIC = - 2 Log L\left( {\underline {{\Theta }}} \right) + 2k,\quad BIC = - 2 Log L\left( {\underline {{\Theta }}} \right) + kLog\left[ n \right]. $$

where, $\underset{\_}{\Theta }$: the vector of distribution parameters, $L\left(\underset{\_}{\Theta }\right)$: the likelihood function, $k$: the number of estimates, *n:* the data size. The small value of -2LL, AIC and BIC mean good-fit distribution. Table 7 shows the result of comparison between our new distribution and other distribution under classical statistics. The result in Table 7 shows our new distribution is better for this data than its classical version in two applications because all goodness of fit tests having smaller values in neutrosophic version than the classical version.

**Table 7:** The result of comparison between our new distribution $NBS({\alpha }_{N},{\beta }_{N})$ and $BS\left(\alpha ,\beta \right)$.

Application	Distribution	MLE	− 2LL	AIC	BIC
Application 1	$NBS({\alpha }_{N},{\beta }_{N})$	${\alpha }_{N}=\left(\mathrm{0.0019,0.0020}\right)$ ${\beta }_{N}=(\mathrm{0.0044,0.0047})$	(2929.146,2999.523)	(5860.827,5897.769)	(5864.169,5904.826)
	$BS\left(\alpha ,\beta \right)$	$\alpha =0.0019$ $\beta =0.0503$	11,939.523	23,855.62	23,887.02
Application 2	$NBS({\alpha }_{N},{\beta }_{N})$	${\alpha }_{N}=\left(\mathrm{0.0626,0.0456}\right)$ ${\beta }_{N}=(\mathrm{0.7583,7546})$	(4403.694,4599.646)	(4407.694,4503)	(4409.474,4505.427)
	$BS\left(\alpha ,\beta \right)$	$\alpha =0.0528$ $\beta =0.7562$	4671.254	4675.254	4677.035

## Conclusion

A new distribution introduced which called neutrosophic Birnbaum–Saunders distribution and denoted by $NBS({\alpha }_{N},{\beta }_{N})$. some statistical properties such as neutrosophic probability density function, neutrosophic cumulative distribution function, neutrosophic hazard function, neutrosophic mean, mode, median, variance, moment of origin, moment generating function, characteristic function, quantile function, cumulant generating function, order statistic and entropies. The neutrosophic maximum likelihood estimators and neutrosophic Bayesian estimators are derived. The simulation study is performed to study the behavior of different estimators at different parameter values and different sample sizes. Also, compare between neutrosophic Birnbaum-Saunders distribution and its classical version using statistical criteria such as bias, MSE, AIC and ACL. Finally, real data of the melting point of alloy and real data of the lifetime of batteries are used to show the validity of $NBS({\alpha }_{N},{\beta }_{N})$ in real life. Also, compare the performance of $NBS({\alpha }_{N},{\beta }_{N})$ and $BS\left(\alpha ,\beta \right)$ on this data using AIC, BIC and − 2LL, which shows the good performance of $NBS({\alpha }_{N},{\beta }_{N})$ than $BS\left(\alpha ,\beta \right).$ The neutrosophic field has more points need more and more search so, in future we try to introduce more netrosophic distribution to used solve and describe more real life applications also we will try to introduce many researches in different points in neutrosophic statistics.

## Data Availability

The data is given in the paper.

## References

[1] Birnbaum , Z. W. & Saunders , S. C. in *Journal of Applied Probability* 319–327 (1969a).

[2] Johnson N, Kotz S, Balakrishnan N (1995). Continuous Univariate Distributions.

[3] Owen WJ, Padgett WJ (2000). Birnbaum–Saunders accelerated life model. IEEE Trans. Reliab..

[4] Guiraud P, Leiva V, Fierro R (2009). A non-central version of the Birnbaum–Saunders distribution for reliability analysis. IEEE Trans. Reliab..

[5] Ho JW (2012). Parameter estimation for the Birnbaum–Saunders distribution under an accelerated degradation test. Eur. Ind. Eng..

[6] Leiva V, Barros M, Paula GA, Galea M (2007). Influence diagnostics in log-Birnbaum–Saunders regression models with censored data. Comput. Stat. Data Anal..

[7] Leiva V, Barros M, Paula GA, Sanhueza A (2008). Generalized Birnbaum–Saunders distributions applied to air pollutant concentration. Environmetrics.

[8] Leiva V, Sanhueza A, Angulo JM (2009). A length-biased version of the Birnbaum–Saunders distribution with application in water quality. Stoch. Env. Res. Risk Assess..

[9] Leiva V, Vilca F, Balakrishnan N, Sanhueza A (2010). A skewed sinh-normal distribution and its properties and application to air pollution. Commun. Stat. Theory Methods.

[10] Leiva V, Athayde E, Azevedo C, Marchant C (2011). Modeling wind energy flux by a Birnbaum–Saunders distribution with unknown shift parameter. J. Appl. Stat..

[11] Leiva V, Ponce MG, Marchant C, Bustos O (2012). Fatigue statistical distributions useful for modeling diameter and mortality of trees. Colomb. J. Stat..

[12] Barros M, Paula GA, Leiva V (2008). A new class of survival regression models with heavy-tailed errors: Robustness and diagnostics. Lifetime Data Anal..

[13] Bhatti CR (2010). The Birnbaum–Saunders autoregressive conditional duration model. Math. Comput. Simul..

[14] Vilca F, Sanhueza A, Leiva V, Christakos G (2010). An extended Birnbaum–Saunders model and its application in the study of environmental quality in Santiago Chile. Stoch. Env. Res. Risk Assess..

[15] Vilca F, Santana L, Leiva V, Balakrishnan N (2012). Estimation of extreme percentiles in Birnbaum–Saunders distributions. Comput. Stat. Data Anal..

[16] Paula GA, Leiva V, Barros M, Liu S (2012). Robust statistical modeling using the Birnbaum–Saunders t-distribution applied to insurance. Appl. Stoch. Model. Bus. Ind..

[17] Ferreira M, Gomes MI, Leiva V (2012). On an extreme value version of the Birnbaum–Saunders distribution. Revstat-Stat. J..

[18] Marchant C, Bertin K, Leiva V, Saulo H (2013). Generalized Birnbaum–Saunders kernel density estimators and an analysis of financial data. Comput. Stat. Data Anal..

[19] Marchant C, Leiva V, Cavieres MF, Sanhueza A (2013). Air contaminant statistical distributions with application to PM_10_ in Santiago, Chile. Rev. Environ. Contam. Toxicol..

[20] Bhattacharyya GK, Fries A (1982). Fatigue failure models—Birnbaum–Saunders versus inverse Gaussian. IEEE Trans. Reliab..

[21] Desmond AF (1986). On the relationship between two fatigue-life models. IEEE Trans. Reliab..

[22] Mann NR, Schafer RE, Singpurwalla ND (1974). Methods for Statistical Analysis of Reliability and Life Data.

[23] Kundu D, Kannan N, Balakrishnan N (2008). On the hazard function of Birnbaum–Saunders distribution and associated inference. Comput. Stat. Data Anal..

[24] Bebbington M, Lai CD, Zitikis R (2008). A proof of the shape of the Birnbaum–Saunders hazard rate function. Math. Sci..

[25] Gupta RC, Akman HO (1997). Estimation of critical points in the mixture of inverse Gaussian distribution. Stat. Pap..

[26] Smarandache F (2010). Neutrosophic logic-a generalization of the intuitionistic fuzzy logic. Multispace Multistruct. Neutrosophic Transdiscipl. (100 Collect. Papers Sci.).

[27] Smarandache, F. *Introduction to Neutrosophic Statistics, Sitech and Education Publisher, Craiova* 123 (Romania-Educational Publisher, 2014).

[28] Chen J, Ye J, Du S (2017). Scale effect and anisotropy analyzed for neutrosophic numbers of rock joint roughness coefficient based on neutrosophic statistics. Symmetry.

[29] Aslam M (2018). A new sampling plan using neutrosophic process loss consideration. Symmetry.

[30] Nayana, B. M., Anakha, K. K., Chacko, V. M., Aslam, M. & Albassam, M. A new neutrosophic model using DUS-Weibull transformation with application. *Complex Intell. Syst.*, 1–10 (2022).

[31] Alhabib R, Ranna MM, Farah H, Salama A (2018). Some neutrosophic probability distributions. Neutrosophic Sets Syst..

[32] Alhasan KH, Smarandache F (2019). Neutrosophic Weibull distribution and neutrosophic family Weibull distribution. Neutrosophic Sets Syst..

[33] Patro SK, Smarandache F (2016). The neutrosophic statistical distribution, more problems, more solutions. Neutrosophic Sets Syst..

[34] Zeina MB (2020). Neutrosophic event-based queueing model. Int. J. Neutrosophic Sci..

[35] Zeina MB (2020). Erlang service queueing model with neutrosophic parameters. Int. J. Neutrosophic Sci..

[36] Alhabib R, Salama AA (2020). The neutrosophic time series-study its models (linear-logarithmic) and test the coefficients significance of its linear model. Neutrosophic Sets Syst..

[37] Alhabib R, Salama AA (2020). Using moving averages to pave the neutrosophic time series. Int. J. Neutrosophic Sci..

[38] Cruzaty LEV, Tomalá MR, Gallo CMC (2020). A neutrosophic statistic method to predict tax time series in ecuador. Neutrosophic Sets Syst..

[39] Bisher M, Hatip A (2021). Neutrosophic random variables. Neutrosophic Sets Syst..

[40] Granados C (2021). New results on neutrosophic random variables. Neutrosophic Sets Syst..

[41] Granados C, Sanabria J (2021). On independence neutrosophic random variables. Neutrosophic Sets Syst..

[42] Kandasamy, W. B. V. & Smarandache, F. Neutrosophic rings (2006).

[43] Salama A, Sharaf Al-Din A, Abu Al-Qasim I, Alhabib R, Badran M (2020). Introduction to decision making for neutrosophic environment study on the Suez Canal Port. Neutrosophic Sets Syst..

[44] Kamaci H (2020). Neutrosophic cubic Hamacher aggregation operators and their applications in decision making. Neutrosophic Sets Syst..

[45] Olgun, N. & Hatip, A. The Effect of the Neutrosophic Logic on The Decision Making, in *Quadruple Neutrosophic Theory and Applications*, (Pons Editions Brussels, Belgium, 2020).

[46] Sahin R (2014). Neutrosophic hierarchical clustering algoritms. Neutrosophic Sets Syst..

[47] Shahzadi G, Akram M, Saeid AB (2017). An application of single-valued neutrosophic sets in medical diagnosis. Neutrosophic Sets Syst..

[48] Ejaita OA, Asagba P (2017). An improved framework for diagnosing confusable diseases using neutrosophic based neural network. Neutrosophic Sets Syst..

[49] Chakraborty A, Banik B, Mondal SP, Alam S (2020). Arithmetic and geometric operators of pentagonal neutrosophic number and its application in mobile communication service based MCGDM problem. Neutrosophic Sets Syst..

[50] Sahin M, Olgun N, Uluçay V, Kargon A, Smarandache F (2017). A new similarity measure based on falsity value between single valued neutrosophic sets based on the centroid points of transformed single valued neutrosophic numbers with applications to pattern recognition. Neutrosophic Sets Syst..

[51] Lotfy MM, ELhafeez S, Eisa M, Salama AA (2015). Review of recommender systems algorithms utilized in social networks based e-learning systems & neutrosophic system. Neutrosophic Sets Syst..

[52] Nabeeh NA, Smarandache F, Abdel-Basset M, El-Ghareeb HA, Aboelfetouh A (2017). An integrated neutrosophic topsis approach and its application to personnel selection: A new trend in brain processing and analysis. IEEE Access.

[53] https://reference.wolfram.com

[54] Cover TM, Thomas JA (2005). Elements of Information Theory.

[55] Rényi, A. On measures of entropy and information. In* Proceedings of the 4th Fourth Berkeley Symposium on Mathematical Statistics and Probability; University of California Press: Berkeley,CA, USA* 547–561 (1961).

[56] Tsallis C (1988). Possible generalization of Boltzmann–Gibbs statistics. J. Stat. Phys..

[57] Lawless, J. F. *Statistical Models and Methods for Lifetime Data*. (John Wiley and Sons, 2003).

[58] Geman S, Geman D (1984). Stochastic relaxation, Gibbs distributions and the Bayesian restoration of images. IEE Transaction on Pattern Analysis and Machine Intelligent.

[59] Smith A, Roberrs G (1993). Bayesian computation via the Gibbs sampler and related Markov chain Monte Carlo methods. Journal of Reliability & Statistical Society.

[60] Metropolis N, Rosenbluth AW, Rosenbluth AH, Teller AH, Teller E (1953). Equations of state calculations by fast computing machine. Journal of Chemistry Physics.

[61] Hastings WK (1970). Monte Carlo sampling methods using Markov chains and their applications. Biometrika.

[62] R Core Team. R: A Language and Environment for Statistical Computing. R Foundation for Statistical Computing, Vienna, Austria (2023).

[63] Rao GS (2023). Neutrosophic log-logistic distribution model in complex alloy metal melting point applications. Int. J. Comput. Intell. Syst..

[64] Aslam M (2021). A new goodness of fit test in the presence of uncertain parameters. Complex Intell. Syst..

